# Genome-wide survey and expression analysis of the *bHLH-PAS* genes in the amphioxus *Branchiostoma floridae* reveal both conserved and diverged expression patterns between cephalochordates and vertebrates

**DOI:** 10.1186/2041-9139-5-20

**Published:** 2014-06-03

**Authors:** Kun-Lung Li, Tsai-Ming Lu, Jr-Kai Yu

**Affiliations:** 1 Institute of Cellular and Organismic Biology, Academia Sinica, 128 Academia Road, Section 2, Nankang, Taipei 11529, Taiwan; 2 Institute of Oceanography, National Taiwan University, Taipei 10617, Taiwan

**Keywords:** Amphioxus, bHLH-PAS transcription factors, *Branchiostoma floridae*, Embryonic development, Molecular phylogeny

## Abstract

**Background:**

The bHLH-PAS transcription factors are found in both protostomes and deuterostomes. They are involved in many developmental and physiological processes, including regional differentiation of the central nervous system, tube-formation, hypoxia signaling, aromatic hydrocarbon sensing, and circadian rhythm regulation. To understand the evolution of these genes in chordates, we analyzed the *bHLH-PAS* genes of the basal chordate amphioxus (*Branchiostoma floridae*).

**Results:**

From the amphioxus draft genome database, we identified ten *bHLH-PAS* genes, nine of which could be assigned to known orthologous families. The tenth *bHLH-PAS* gene could not be assigned confidently to any known bHLH family; however, phylogenetic analysis clustered this gene with arthropod *Met* family genes and two spiralian bHLH-PAS-containing sequences, suggesting that they may share the same ancestry. We examined temporal and spatial expression patterns of these *bHLH-PAS* genes in developing amphioxus embryos. We found that *BfArnt*, *BfNcoa*, *BfSim*, and *BfHifα* were expressed in the central nervous system in patterns similar to those of their vertebrate homologs, suggesting that their functions may be conserved. By contrast, the amphioxus *BfAhr* and *BfNpas4* had expression patterns distinct from those in vertebrates. These results imply that there were changes in gene regulation after the divergence of cephalochordates and vertebrates.

**Conclusions:**

We have identified ten *bHLH-PAS* genes from the amphioxus genome and determined the embryonic expression profiles for these genes. In addition to the nine currently recognized *bHLH-PAS* families, our survey suggests that the *BfbHLHPAS-orphan* gene along with arthropod *Met* genes and the newly identified spiralian bHLH-PAS-containing sequences represent an ancient group of genes that were lost in the vertebrate lineage. In a comparison with the expression patterns of the vertebrate *bHLH-PAS* paralogs, which are the result of whole-genome duplication, we found that although several members seem to retain conserved expression patterns during chordate evolution, many duplicated paralogs may have undergone subfunctionalization and neofunctionalization in the vertebrate lineage. In addition, our survey of amphioxus *bHLH-PAS* gene models from genome browser with experimentally verified cDNA sequences calls into question the accuracy of the current *in silico* gene annotation of the *B. floridae* genome.

## Background

The bHLH-PAS proteins are metazoan transcription factors characterized by the presence of a basic-helix-loop-helix (bHLH) domain and a Per-ARNT-Sim (PAS) domain. The bHLH domain is composed of an N-terminal DNA-binding basic (b) region followed by two α-helices connected by a loop (HLH) [1]. The HLH region promotes dimerization, which enables the formation of homodimeric or heterodimeric bHLH protein complexes, and the basic regions of the complexes recognize specific response elements on DNA [2]. Metazoan bHLH transcription factors are grouped into 45 families and 6 higher-order groups from A to F [3,4]. The PAS domain is named for the Period (Per, from fruit fly), Aryl hydrocarbon receptor nuclear translocator (ARNT, from human), and Single-minded (Sim, from fruit fly) proteins, in which the homology of this domain was first discovered [5,6]. PAS domains consist of approximately 275 amino acids and can be subdivided into two PAS repeats: PAS A and PAS B [7,8]. PAS domains not only promote heterodimerization but also have other functions, including ligand binding and interaction with non-PAS proteins (reviewed in [5,7]). PAS domain-containing proteins are present in Bacteria, Archaea, and Eukarya [8].

Genes encoding proteins with both bHLH and PAS domains (*bHLH-PAS* genes) are believed to have an ancient origin, as they exist throughout metazoa, from humans to basal animals, such as the demosponge *Amphimedon queenslandica*[4] and the placozoa *Trichoplax adhaerens*[9,10]. Most bHLH-PAS families have been placed in the higher-order group C based on their molecular phylogeny and DNA-binding specificity, but previous analyses were equivocal on whether these bHLH-PAS proteins form a monophyletic group [3,4].

The bilaterian bHLH-PAS protein complement is stable in terms of the number of families; model protostomes and vertebrates share nine bHLH-PAS families [3,11], as follows: Nuclear receptor coactivator (NCOA/SRC), Circadian locomotor output cycles kaput (Clock), Aryl hydrocarbon receptor nuclear translocator (ARNT), Brain and muscle ARNT-like (Bmal/cycle), Aryl hydrocarbon receptor (AHR), neuronal PAS domain protein 4 (NPAS4/dysfusion), Single-minded (Sim), Trachealess (Trh in fly and NPAS1/3 in vertebrates), and Hypoxia inducible factor (HIF). These bHLH-PAS proteins are involved in various important developmental and/or physiological processes, including the regional specification or differentiation of the central nervous system (CNS) (Sim family in fly and mammals; Npas1 and Npas3 in mammals) [5,7], tube-formation (trh and dys in fly; Npas1 and Npas3 in mouse) [12-14], hypoxia signaling (HIF family) [15,16], aromatic hydrocarbon sensing (AHR in mammals) [17,18], and circadian rhythm (Clock and Bmal/cycle families) [19,20]. However, another protein family, the Methoprene-tolerant (Met) proteins, also contains bHLH and PAS domains [21], but to date this family has no well-characterized ortholog in non-arthropod organisms.

The evolution of bHLH-PAS protein functions, however, remains poorly understood. Certain functions appear to be highly conserved between protostomes and vertebrates; for example, genes of the HIF family participate in hypoxia responses in diverse organisms (reviewed in [15]). By contrast, some orthologs play very different roles; for example, whereas mouse *Npas4* is related to neural activity in the CNS [22-24], its homolog in fly, *dysfusion*, is primarily required for regulating the development of tracheal fusion cells [25,26].

Comparative genomic studies have shown that the vertebrate lineage has undergone at least two rounds of whole-genome duplication [27,28]. As such, it is possible to deduce ancestral gene function and functional divergence in different lineages by comparing vertebrate genes to those of organisms that did not undergo duplication (‘pre-duplicated’ genes). Such organisms include the amphioxus (*Branchiostoma floridae*), which has recently been suggested to be the basal chordate clade [28-31]. Studies on amphioxus have been facilitated by the sequencing of its genome and by the available cDNA and EST resources [28,32-34]. Previous surveys based on gene models predicted the existence of nine families of *bHLH-PAS* genes in amphioxus, but experimental validation of transcripts and the expression patterns of these genes were lacking [4,11]. To verify the *bHLH-PAS* gene complement in the amphioxus genome, we manually annotated amphioxus *bHLH-PAS* genes from the draft genome of *B. floridae* using available cDNA sequences, and we further examined the developmental expression patterns of these *bHLH-PAS* genes. We also compared our *bHLH-PAS* cDNA sequences to corresponding gene models, revealing frequent inaccuracies in the original models.

## Methods

### Identification of *bHLH-PAS* genes in the amphioxus genome and procurement of *bHLH-PAS* cDNA sequences

To identify amphioxus *bHLH-PAS* genes, sequences of representative human bHLH-PAS proteins were used to perform separate searches of the *B. floridae* genome [28,32] and the amphioxus cDNA and EST database [33]. The family names, protein names, and accession numbers of human proteins used are: NCOA (SRC): NCOA2 [Swiss-Prot: Q15596]; Clock: CLOCK [Swiss-Prot: O15516.1]; ARNT: ARNT [Swiss-Prot: P27540.1]; Bmal/cycle: BMAL1 [Swiss-Prot: O00327.2]; AHR: AHR [Swiss-Prot: P35869.2]; NPAS4/dysfusion: NPAS4 [Swiss-Prot: Q8IUM7.1]; Sim: SIM1 [Swiss-Prot P81133.2]; Trh: NPAS3 [Swiss-Prot: Q8IXF0.1]; and HIF: HIF1A [Swiss-Prot: Q16665.1].

We performed BLASTp searches of the *B. floridae* filtered gene models database via the US Department of Energy Joint Genome Institute genome browser [35]. The resulting protein models were used for BLASTp searches of the National Center for Biotechnology Information (NCBI) non-redundant protein sequences (nr) database to test the reciprocal best-hit relationship [36]; this relationship was used to initially assign each protein model to a particular family (Table 1). These families were named as described previously [3,36], with the exceptions of NCOA (former SRC), Bmal/cycle (former Bmal) and NPAS4/dysfusion.

**Table 1:** ***B. floridae bHLH-PAS *** **gene models and identified cDNA clones**

**Family name**	**Protein ID (Expect value)**	**cDNA cluster ID (Expect value)**	**Accession number**	**cDNA length (nt)**	**Full length coding sequence**	**Best hit human protein**	**Name given after phylogenetic analyses**	**Related reports**^**a**^
NCOA (SRC)	212110 (1.42e^-62^)	N/A	KC305624	3,004^b^	Yes (1911 nt)	NCOA2 (Q15596.2)	*BfNcoa*	Bb, *NCOA* (JN831351.1) [58]
ARNT	124387 (4.79e^-179^)	01670 (1e^-63^)	KC305625	3,418^b^	Yes (2139 nt)	ARNT2 (AAG15310.1)	*BfArnt*	
orphan^c^	117200; 125569	16184	KC305626	2,659^b^	Yes (2418 nt)	BMAL1e (BAA19938.1)	*BfbHLHPAS-orphan*	
Clock	63636 (3.72e^-137^); 63642 (6.95e^-109^)	N/A	KC305627	2,425^b^	No (2343 nt)	CLOCK (O15516.1)	*BfClock*	
Bmal/cycle	110703 (0e^0^); 96140 (8.13e^-77^)	N/A	KC305628	1,782^b^	No (1782 nt)	MOP3 (BMAL1) (AAC51213.1)	*BfBmal*	Bl, *amphiBmal*[60]
AHR	235977 (4.04e^-79^); 98840 (5.68e^-14^)	N/A	KC305629	1,047^b^	No (915 nt)	AHR (BAA03857.1)	*BfAhr*	
NPAS4/dysfusion	121518 (3.54e^-66^)	N/A	KC305630	2,529	No (2526 nt)	NPAS4 (NP_849195.2)	*BfNpas4*	
Sim	265033 (0e^0^)	N/A	KC305631	999	No (996 nt)	SIM1 (P81133.2)	*BfSim*	Bf, *AmphiSim* (AJ506161.1) [55]
Trh	220692 (1.75e^-53^); 220681 (4.62e^-48^); 163191 (1.73e^-24^); 220639 (4.06e^-23^) and others	N/A	KC305632	1,176^b^	No (1104 nt)	NPAS3 transcript variant 1 (AAO17043.1)	*BfNpas1/3*	
HIF	208339 (5.04e^-114^); 208408 (1.73e^-105^)	13696 (5e^-55^); 04181 (4e^-51^); 00128 (3e^-46^)	KC305633	4,514^b,d^;	Yes (2676 nt)	HIF1A (NP_851397.1)	*BfHifα*	Bj, *Hifα* (HM188448- HM188451) [59]
			KC305634	2,809^b,d^	Yes (2589 nt)	HIF1A (NP_851397.1)	*BfHifα*	

^a^Expressions in amphioxus reported. Bb, *Branchiostoma belcheri*; Bl, *B. lanceolatum*; Bf, *B. floridae*; Bj, *B. japonicum* (*B. belcheri tsingtauense* in their article). ^b^Obtained cDNA sequences do not agree with Joint Genome Institute gene models. ^c^See Results for its obtainment. ^d^The long isoform (KC305633) was from cDNA cluster 00128; the short isoform (KC305634) was from 13696. These two forms correspond to the same Joint Genome Institute gene model and may be different products of the same gene. See Results for detail. N/A, not applicable; nt, nucleotides.

For tBLASTn searches of the cDNA and EST database, only searches using ARNT and HIF led to EST sequences that gave a reciprocal best-hit relationship. Sequencing of these cDNA clones (bfne124n01 for *BfArnt*; bfad013f17 and bfad009d19 representing two isoforms for *BfHifα*) confirmed that they represent the orthologs of the query genes. Searches using other bHLH-PAS proteins gave no reliable results.

The cDNA of gene models without EST clones was amplified by PCR using a cDNA library constructed in the pBluescript vector [37]. PCR was performed with gene-specific primer sets using the Expand High Fidelity^PLUS^ PCR System (Roche, Basel, Switzerland). PCR products were ligated into the pGEM®-T easy vector (Promega, Fitchburg, Wisconsin, USA), amplified, and then sequenced. The primers and sizes of the cDNA fragments obtained by PCR amplification are listed in Additional file 1: Table S1.

### Domain comparison and phylogenetic analysis

Predicted amphioxus protein sequences were used to search the Pfam database [38] for conserved domain annotation. The sequences of bHLH-PAS proteins from other species used for comparison and phylogenetic analysis were retrieved from the NCBI protein database with the exception of sea urchin Hifα, which is an unpublished sequence from Dr Yi-Hsien Su’s laboratory. To infer evolutionary relationships, a concatenated alignment of bHLH, PAS A, and PAS B domains of all obtained protein sequences was built with the ClustalW algorithm [39] of the BioEdit program (version 7.0.5.3) [40]. Phylogenetic analysis using the neighbor-joining method was performed with MEGA5 [41]. The results were further examined using the maximum-likelihood method with RAxML-HPC BlackBox (8.0.9) via the CIPRES Science Gateway [42,43] with the same alignment.

To further investigate the phylogenetic affinity of BfbHLHPAS-orphan and arthropod Met proteins, we used a BfbHLHPAS-orphan peptide sequence to perform BLASTp searches onto the Genome Browser for *Branchiostoma belcheri*, B.belcheri_HapV2_proteins database [44,45]. We found a predicted sequence (203360_PRF0, denoted as Bb_orphan in this study) that was almost identical to our ‘Bf orphan’ protein (high BLAST score, expect value = 0.0). We also searched the newly available genome data of *Capitella teleta* (Annelida) and *Lottia gigantea* (Mollusca) [46] and retrieved three highest-score sequences from each genome. Phylogenetic analyses of these sequences were performed.

### Animal collection

Adult amphioxus animals were collected in Tampa Bay, Florida, USA, during the summer breeding season. Gametes were obtained by electric stimulation. Fertilization and culturing of the embryos were carried out as previously described [47]. Amphioxus embryos were staged according to Hirakow and Kajita [48,49], and neurula-stage embryos were further divided into finer stages according to Lu *et al*. [50].

### Quantitative PCR

To examine the expression level of each *bHLH-PAS* gene at representative embryonic stages and in adults, cDNA samples were prepared as previously described [51]. To examine the expression of circadian clock-related genes in amphioxus cerebral vesicle, we raised post-metamorphosis amphioxus juveniles in a 14:10-h light/dark cycle for more than two weeks. Approximately 3.5 hours after light on/off, the animals were sacrificed, and total RNA of the anterior body part (approximately 10% of body length) was isolated using the RNeasy Micro kit (Qiagen, Hilden, Germany) and then reverse transcribed using the iScript cDNA synthesis kit (Bio-Rad, Hercules, California, USA) as previously described [51]. We also designed quantitative PCR (Q-PCR) primers based on the gene model of *BfPeriod* (the Joint Genome Institute (JGI) genome browser, protein ID: 67319) to determine whether expression of circadian clock-related genes follows circadian oscillation. The Q-PCR primers used are listed in Additional file 2: Table S2. The Q-PCR analysis was performed on a Roche LightCycler 480 machine using the LightCycler 480 SYBR Green I Master system (Roche). The expression level of each gene was normalized to the 18S rRNA level of each sample. All products of Q-PCR reactions were verified by sequencing.

### *In situ* hybridization and image acquisition

To synthesize riboprobes, cDNA fragments were amplified as templates. For *BfNcoa*, *BfAhr*, and *BfSim*, cDNA fragments ligated into the pGEM®-T easy vector (Promega) were directly amplified with T7 and SP6 primers. For *BfNpas4*, *BfArnt*, and *BfHifα*, we designed primers to amplify appropriate fragments as templates. Antisense or sense digoxigenin (DIG)-labeled riboprobes were synthesized using DIG RNA labeling mix (Roche) with T7 or SP6 RNA polymerase (Promega), depending on the insert orientation. Sense riboprobes were synthesized as negative controls for all the genes we examined. Whole-mount *in situ* hybridization on amphioxus embryos was performed as previously described [50]. To detect *BfHifα* expression in amphioxus juveniles, fixed samples (approximately 1 cm long) were transverse-sectioned (16 μm thick) on a cryostat (CM3050s, Leica, Wetzlar, Germany), thaw-mounted on glass slides (MAS-GP type A coated glass slide, Matsunami, Kishiwada City, Japan) and stored at -20°C. *In situ* hybridization of cryosection samples was performed as for whole-mount samples, but with the following modification: cryosections were thawed, dried at 37°C for 1 h, and washed in phosphate-buffered saline with Tween 20 (PBST) three times; proteinase K treatment was omitted and the samples were rinsed in 0.1 M triethanolamine before proceeding with the acetic anhydride treatment. The rest of the procedure was the same as the described *in situ* hybridization method. Images of embryos were taken using a Zeiss Axio Imager A1 microscope with a Zeiss AxioCam MRc CCD camera, and images of cryosections were taken using a Leica Z16APO microscope with a Leica DFC 300FX camera. Double-fluorescent *in situ* hybridization was performed essentially as described previously [51]. Dinitrophenol (DNP)-labeled *BfSim* antisense riboprobe was synthesized using Label IT® nucleic acid labeling reagents (Mirus, Madison, Wisconsin, USA), and DIG-labeled antisense riboprobe for the pan-neural marker *AmphiElav/Hu* was synthesized as described [50]. We used anti-DIG-POD and anti-DNP-POD antibodies (Roche) to detect the riboprobes, and then used the TSA Plus Cyanine 3 & Fluorescein system (PerkinElmer, Waltham, Massachusetts, USA) to amplify the fluorescent signals. Samples were photographed using a Leica TCS-SP5 confocal microscope. Adobe Photoshop CS4 was used to minimally adjust the brightness of photographs, as well as to construct montage images of whole larvae from multiple photographs.

### Comparisons of obtained cDNA sequences to corresponding genomic scaffolds and gene models

The obtained cDNA sequences were used to perform BLASTn searches against the *B. floridae* draft genome (Bf_v1.0 unmasked assembly), to determine the relationship between the cDNA, the genomic scaffolds, and the corresponding gene models. The ambiguous result of *BfBmal*-scaffold 279 was further analyzed with the Spidey program [52,53]. Similar amphioxus genomic scaffolds or scaffold regions were compared via Blast 2 sequences (NCBI).

## Results

### Identification of amphioxus *bHLH-PAS* genes

In this study, more than 18 gene models were recovered in the BLAST searches of the *B. floridae* filtered gene models database. In Table 1, models having reciprocal best-hit relationships and including the bHLH and/or PAS domains were recorded and initially assigned to a particular family, and these models were used in subsequent investigations. Because of the high allelic polymorphism of the amphioxus genome [28], we found many redundant gene models in the current assembly. To verify the existence and the expression of the identified gene models, we searched the cDNA and EST database or used PCR amplification to find supporting evidence. We also used a previously reported gene model (117200) [4] to query the cDNA and EST database and recovered the cDNA cluster 16184 (clone bfeg037n07) with an expect-value of 1e^-76^. This cDNA clone was sequenced and analyzed. It corresponds to two models (117200 and 125569) but could not be assigned to any known bHLH family. Thus, we provisionally named it *BfbHLHPAS-orphan*. In sum, by PCR cloning and searching the cDNA and EST library we identified 10 amphioxus *bHLH-PAS* genes corresponding to 11 cDNA sequences (NCBI accession numbers [GenBank:KC305624 to KC305634]; Table 1).

We used these cDNAs to perform BLASTx searches on the NCBI nr human protein database; as Table 1 shows, all cDNA sequences, except the *BfbHLHPAS-orphan*, hit the initial query human proteins or their paralogs within the same family (ARNT/ARNT2). This reciprocal best-hit relationship was the first evidence to support the orthology of each family [36]. The assignment of the *BfbHLHPAS-orphan* gene will be discussed in following sections.

### Conserved domains of bHLH-PAS proteins

Based on the sequences of cDNA clones or assemblies, although without the full-length coding sequences of many genes, all of the predicted proteins have conserved bHLH, PAS A, and PAS B domains (Additional file 3: Figure S1). The sequence alignments of the bHLH, PAS A, and PAS B domains of amphioxus and selected human proteins show significant conservation of these protein domains between human and amphioxus (Additional file 4: Figure S2). In addition, the BfHifα protein has a presumed oxygen-dependent degradation domain and C-terminal trans-activation domain (Additional file 3: Figure S1). Within these domains, presumed hydroxylation sites (two proline sites, one asparagine site), which are important in stability and activity regulation, are also conserved (Additional file 5: Figure S3). Predicted proteins from two forms of *BfHifα* cDNA are nearly identical except that the short isoform (‘s’ in Additional files 3 and 5) lacks the N-terminal part of the presumed oxygen-dependent degradation domain including the first presumed hydroxylation target proline.

### Phylogenetic analyses

**Figure 1:**
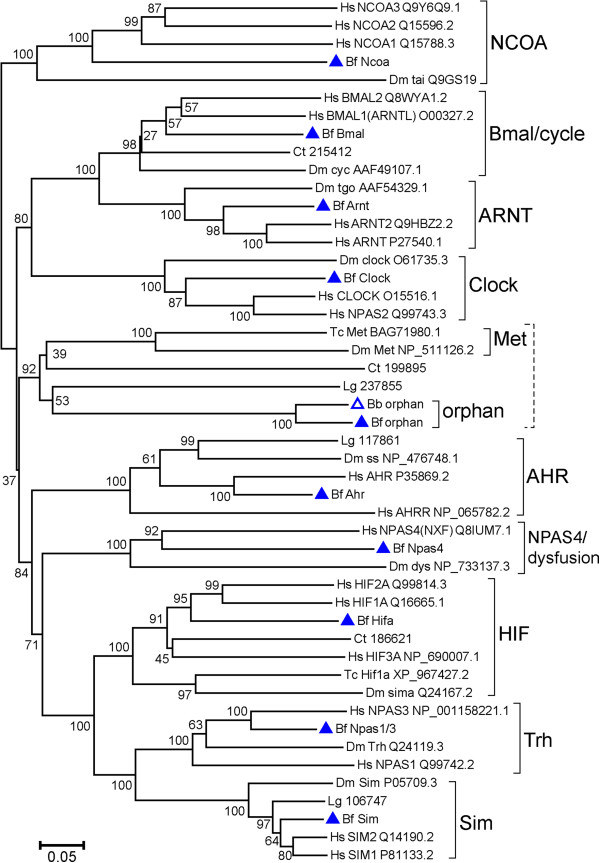
**Phylogenetic analysis of all bHLH-PAS protein families with neighbor-joining method.** The tree is a neighbor-joining bootstrap consensus tree based on a concatenated alignment of bHLH, PAS A, and PAS B domains. The rooting should be considered as arbitrary. Bootstrap support values (as percentages) from 1,000 replicates of each branch are shown. *Branchiostoma floridae* proteins are labeled with filled blue triangles. The BbbHLHPAS-orphan (Bb orphan) from *B. belcheri* is labeled with an open blue triangle. Insect methoprene-tolerant (Met) proteins and spiralian predicted proteins were included because these sequences had high scores when we used the BfbHLHPAS-orphan sequence to perform BLASTp searches. Spiralian sequences are labeled as abbreviations (Ct for *Capitella teleta* and Lg for *Lottia gigantea*) + protein ID on the Joint Genome Institute genome browser. This tree shows that nine amphioxus proteins are grouped into well-known bHLH-PAS families with high bootstrap support (≥98%). Two amphioxus ‘orphan’ proteins, two insect Met proteins, and two spiralian predicted proteins (Ct199895 and Lg237855) form a cluster with a 92% bootstrap support. The tree is drawn to scale, with branch lengths in the same units as those of the evolutionary distances used to infer the phylogenetic tree. The evolutionary distances were computed using the p-distance method and units used are the number of amino acid differences per site. The analysis involved 48 amino acid sequences. All ambiguous positions were removed for each sequence pair. There were a total of 258 positions in the final dataset. Bb, *Branchiostoma belcheri*; Bf, *Branchiostoma floridae*; Ct, *Capitella teleta*; Dm, *Drosophila melanogaster*; Hs, *Homo sapiens*; Lg, *Lottia gigantea*; Tc, *Tribolium castaneum*.

**Figure 2:**
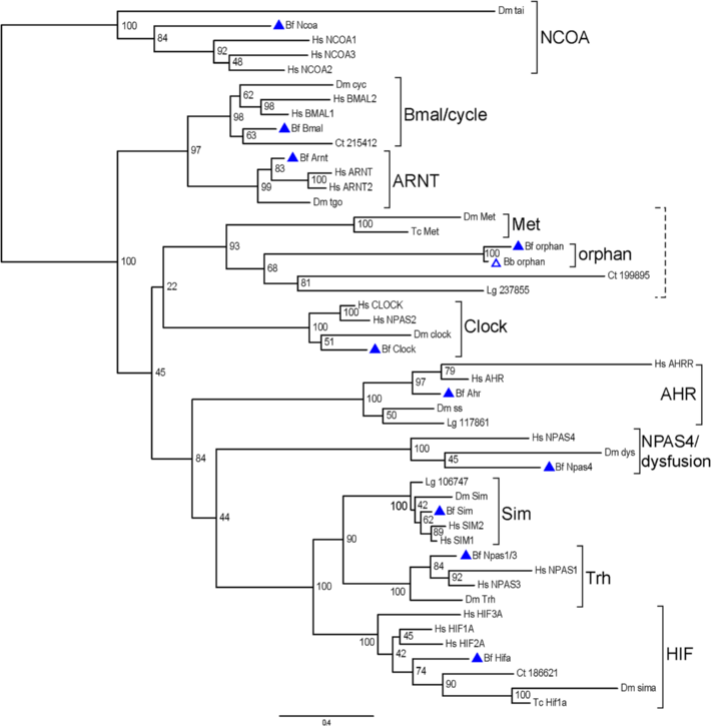
**Phylogenetic analysis of all bHLH-PAS protein families with maximum-likelihood method.** The tree is the best-scoring maximum-likelihood with bootstrap support values of each branch. This tree is based on the same sequence alignment in Figure 1. The rapid bootstrap search was automatically halted after 650 replicates when obtaining stable support values. The rooting should be considered as arbitrary. The LG amino acid substitution model was used. Scale bar: expected changes per site. This tree shows a comparable clustering of sequences as in Figure 1. Abbreviations are as in Figure 1.

We performed phylogenetic analyses with neighbor-joining and maximum-likelihood methods (Figures 1 and 2, respectively) using a concatenated alignment of the bHLH, PAS A, and PAS B domains. The results from both methods showed that nine amphioxus sequences could be clustered into the nine previously recognized families (NCOA, Clock, Bmal/cycle, ARNT, AHR, NPAS4/dysfusion, HIF, Sim, and Trh) with well-supported bootstrap values (neighbor-joining: 98% to 100%; maximum-likelihood: 98% to 100%). Thus, for these nine amphioxus sequences, our initial assignments to each family were supported by the phylogenetic analyses. The BfbHLHPAS-orphan, along with the BbbHLHPAS-orphan from *B. belcheri* genome, did not cluster with the nine known families; instead they were affiliated with arthropod Met sequences and the two spiralian sequences (Ct199895 and Lg237855) with high bootstrap values (neighbor-joining: 92%; maximum-likelihood: 93%). Thus, they may constitute a previously unrecognized *bHLH-PAS* family.

### Temporal expression patterns of *bHLH-PAS* genes

**Figure 3:**
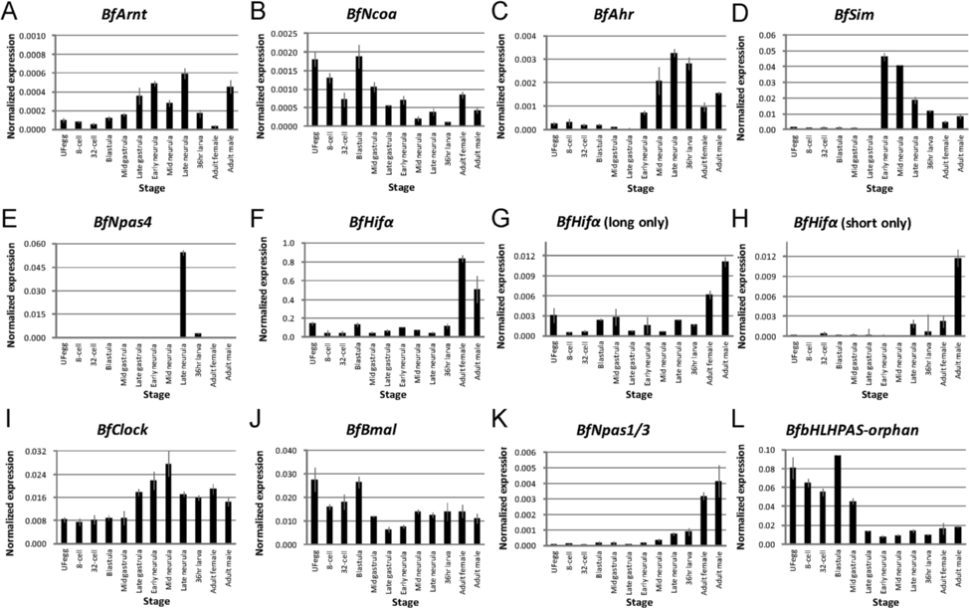
**Relative transcript levels of amphioxus *bHLH-PAS *genes at representative developmental stages. (A-L)** Transcript levels of amphioxus *bHLH-PAS* genes, shown as percentages of those of 18S rRNA. Error bars show the standard deviation of technical replicates. Developmental stage of samples are: unfertilized egg (UFegg), 8-cell morula (8-cell), 32-cell morula (32-cell), Blastula, G3 (Mid gastrula), G5 (Late gastrula), N1 (Early neurula), N2 (Mid neurula), N3 (Late neurula), L2 (36 hr larva), adults (female and male). Because each quantitative PCR primer pair had unequal efficiency in amplification, the resultant relative expression level of different genes or primer pairs may not be directly compared to those of other primer pairs. For *BfHifα*, three primer sets with amplicons on exon 17 to 18 (F), exon 11 to 12 (G), and exon 10 to 12 without exon 11 (H) were used to show the total *BfHifα* expression level and those of different transcript isoforms.

To understand how *bHLH-PAS* genes are expressed in developing amphioxus, we studied the expression levels of all of the identified genes by Q-PCR. Figure 3 shows the temporal expression patterns at representative developmental stages of these *bHLH-PAS* genes. The majority of these genes were not represented in the maternal mRNA; only *BfNcoa*, *BfBmal*, and *BfbHLHPAS-orphan* were represented significantly in maternal mRNA (Figure 3B,J,L). Most of the genes were activated during embryogenesis, but *BfNpas1/3* was not significantly expressed in the embryonic stages that we examined but was expressed significantly in adult animals. We also used Q-PCR primer sets that could differentiate between different *BfHifα* isoforms and found similar expression profiles for these two isoforms (Figure 3F-H).

In addition, homologs of Bmal/cycle and Clock families are known to participate in circadian rhythm regulation; therefore, we further examined the expression levels of *BfBmal* and *BfClock*, as well as that of another presumed ‘clock gene’, *BfPeriod*[54], during the light- or dark-phase of incubation using Q-PCR. We found that while the expression level of *BfPeriod* was significantly higher during the light period, the expression levels of both *BfClock* and *BfBmal* were not significantly different between the light period and the dark period (Additional file 6: Figure S4).

### Spatial expression patterns of *bHLH-PAS* genes

We also determined the spatial expression patterns of *BfArnt*, *BfNcoa*, *BfAhr*, *BfSim*, *BfNpas4*, and *BfHifα* by *in situ* hybridization. However, we could not obtain successful *in situ* hybridization of *BfClock*, *BfBmal*, *BfNpas1/3*, or *BfbHLHPAS-orphan* to show their spatial expression patterns.

**Figure 4:**
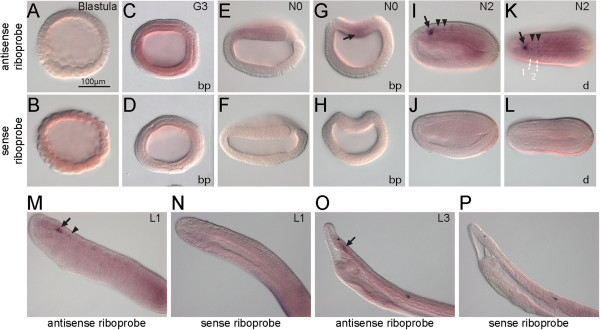
**Expression of *BfArnt*.***In situ* hybridization of *BfArnt* with antisense probe and with sense probe. **(A,B)** No apparent expression is shown at the blastula stage. **(C,D)***BfArnt* is ubiquitously expressed at mid gastrula stage. **(E-H)** Stronger *BfArnt* signals are detected in the dorsal part of the early neurula (arrow in G). **(I-P)** Beginning at mid-neurula stage, some regions of the central nervous system (CNS) specifically express *BfArnt* at a higher level. The CNS region showing the strongest expression (arrows) is next to the first somite, and this region seems to maintain the expression until two-day larva. Patches of weak expression are distributed in specific CNS cell clusters (arrowheads in **I,K,M**), but these patches fade when the embryos reach the larval stage. The scale bar applies to all panels. Blastoporal (bp) views and dorsal views (d) are labeled, and unlabeled panels are lateral views. In E, F, I-L, anterior is to the left; in **M-P**, anterior is to the upper left. Boundaries of somites are depicted in K.

Figure 4 shows the expression of *BfArnt*. It was not significantly expressed during early embryogenesis. At neurula stages, stronger signals were detected in the dorsal part of the embryo and were concentrated in the anterior CNS next to the first somite (Figure 4G,I,K). There was continued strong CNS expression in the cerebral vesicle (arrows in Figure 4I,K,M,O) during subsequent development. There was some weak CNS expression distributed posterior to the cerebral vesicle (arrowheads in Figure 4I,K,M), but these signals faded when the embryos reached the larval stage.

**Figure 5:**
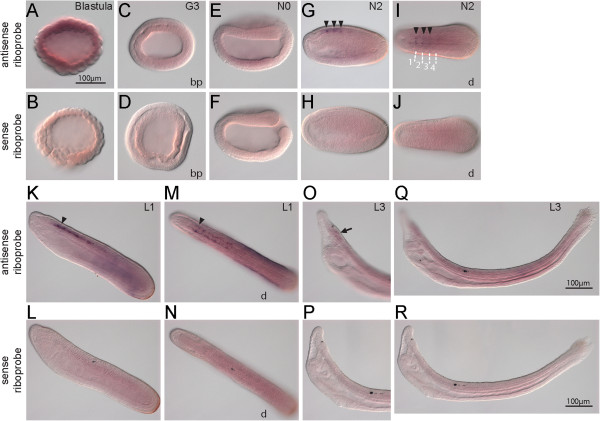
**Expression of *BfNcoa*.***In situ* hybridization of *BfNcoa* with antisense probe and with sense probe. **(A-F)** Ubiquitous expression is shown from blastula to early neurula. **(G-N)** From mid-neurula, tissue-specific signal is detected in some paired cell groups in the anterior central nervous system (arrowheads). **(O,P)** At the larval stage, stronger expression is observed in the cerebral vesicle (arrow), and weaker expression is observed in the neural tube. **(Q,R)** No apparent expression is found in the gut. The scale bar in panel A applies to panels A-P. Blastoporal views (bp) and dorsal views (d) are labeled, and unlabeled panels are lateral views. In E-J, anterior is to the left; in K-R, anterior is to the upper left. Boundaries of somites are depicted in I.

Figure 5 shows the expression of *BfNcoa*. Before the blastula stage, the *BfNcoa* transcripts were distributed ubiquitously (Figure 5A). From N2 stage, tissue-specific expression was detected in some cells inside the CNS (arrowheads, Figure 5G,I). These paired cells were located in the neural tube from the second to fourth somite level, just posterior to the cerebral vesicle. At the early larval stage (L1), strong expression was detected in two rows of cells inside the neural tube (Figure 5K,M); subsequently in the late larval stage (L3), only weak expression was detected in the anterior neural tube (Figure 5O).

**Figure 6:**
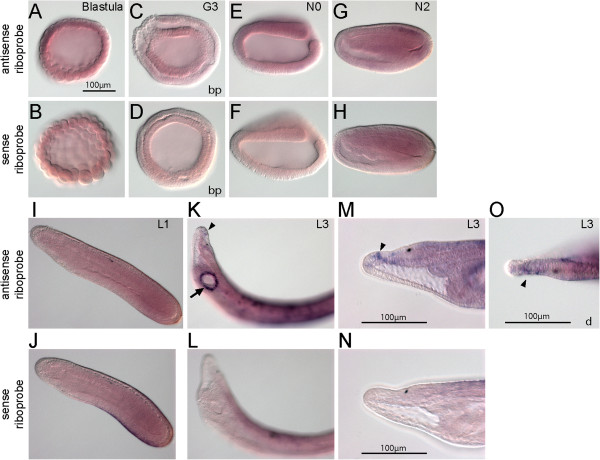
**Expression of *BfAhr*.***In situ* hybridization of *BfAhr* with antisense probe and with sense probe. **(A-J)** Early embryonic and larval stages show no specific expression pattern. **(K-O)** In two-day-old larvae, *BfAhr* is expressed in a circle of cells, two to three cells in width, surrounding the newly opened mouth (arrow in K), and in a few cells located in the epidermis of the rostrum (arrowhead in K,M,O). Most of the rostral *BfAhr*-expressing cells appear to be located on the left side **(O)**. The scale bar in A applies to panels **A-L**. Blastoporal views (bp) and dorsal views (d) are labeled, and unlabeled panels are lateral views. In **E-H** and **M-O**, anterior is to the left; in **I-L**, anterior is to the upper left.

Figure 6 shows the expression of *BfAhr*. No tissue-specific expression was detected from blastula to early larvae (Figure 6A-J). However, in two-day-old larvae, *BfAhr* was specifically expressed in two regions: a circle of cells surrounding the mouth (Figure 6K) and few cells in the epidermis of the rostrum (Figure 6M,O).

**Figure 7:**
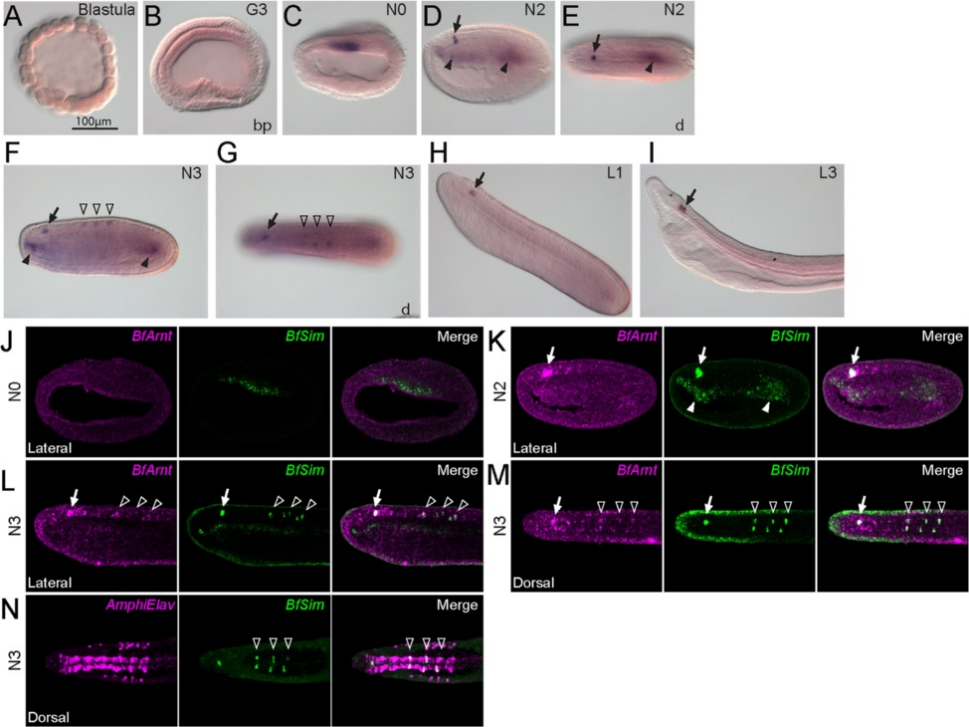
**Expression of *BfSim*. (A-I)***In situ* hybridization of *BfSim* with antisense probe. **(A,B)** Early stages show no expression. **(C)***BfSim* is expressed in a broad band of mesendodermal cells in the early neurula. **(D,E)** In mid-neurula (six somites), expression is localized to three areas: the pharynx roof, the posterior mesendoderm (arrowheads), and a patch of cells in the future cerebral vesicle (arrows). **(F,G)** In the late neurula (≥nine somites), the expression in cerebral vesicle is maintained, and *BfSim* expression was discovered in six clusters of cells, which are paired, within the central nervous system (CNS) (open arrowheads). **(H,I)** In larvae, the expression is maintained in the CNS cells, while the expression in the mesendodermal areas fades. **(J-M)** Double-fluorescent *in situ* hybridization images show the co-localization of *BfSim* and *BfArnt*. **(J)** In the early neurula, *BfArnt* is expressed ubiquitously, but *BfSim* expression is localized to dorsal mesendoderm. **(K)** In the mid-neurula, the *BfSim*-expressing cells in the CNS co-localize with the *BfArnt* expression (arrows); arrowheads show mesendodermal *BfSim* expression. **(L,M)** In the late neurula, the expression in CNS cells is maintained (arrows), and *BfSim* and *BfArnt* are co-expressed in six clusters of cells (open arrowheads). **(N)** The six clusters expressing *BfSim* also express the pan-neural marker *AmphiElav/Hu* (open arrowheads). The scale bar in A applies to panels A-I. Blastoporal views (bp) and dorsal views (d or Dorsal) are labeled, and other panels are lateral views. In C-G and J-N, anterior is to the left; in H and I, anterior is to the upper left.

Our *in situ* hybridization results for *BfSim* (Figure 7) were similar to those of Mazet and Shimeld, published previously [55]. Embryonic *BfSim* expression was first observed in early neurula in the dorsal mesoderm (Figure 7C); subsequently, *BfSim* was also expressed strongly in the forming cerebral vesicle (Figure 7D-I, arrows). In addition, we discovered weak *BfSim* expression in six cell clusters in the late neurula (N3, ≥ nine somites) (open arrowheads in Figure 7), which had not been described previously. Detecting these cells with low *BfSim* expression required a prolonged staining time (over two days). We found that those *BfSim*-expressing cells also expressed *BfArnt* (Figure 7J-M). Additionally, we confirmed that the six *BfSim*-expressing clusters were located within the CNS, based on the co-localization of *BfSim* and the pan-neural marker *AmphiElav/Hu*[56] (Figure 7N).

**Figure 8:**
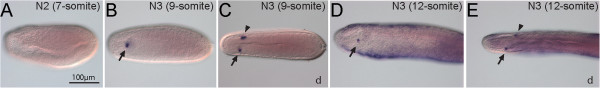
**Expression of *BfNpas4*.***In situ* hybridization of *BfNpas4* with antisense probe. **(A)** No expression is detected in mid-neurula with seven somites. **(B,C)** In the nine-somite late neurula, *BfNpas4* is expressed in two regions; the left region (arrows) is slightly anterior to the right region (arrowhead). **(D,E)** This expression pattern is maintained in the late neurula with 12 somites, and the distance between the two regions increases. The scale bar applies to all panels. Anterior is to the left. A, B, and D are lateral views; C and E are dorsal (d) views.

The expression of *BfNpas4* was detected in the late neurula stage embryo with at least nine somites (Figure 8). *BfNpas4* was expressed in two spots located in both sides of the mesendoderm adjacent to the first somite (Figure 8B). The spot on the left was relatively more anterior than the right one (Figure 8C,E). All other examined stages showed no significant trace of expression, which was consistent with our Q-PCR analysis (Figure 3E). These results suggest that *BfNpas4* is sharply regulated and only expressed within a short time window during development.

**Figure 9:**
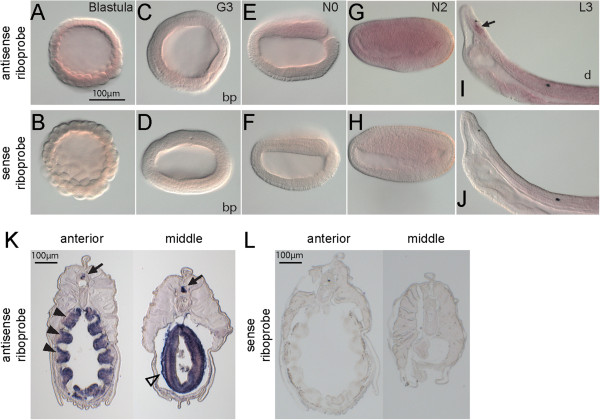
**Expression of *BfHifα*.***In situ* hybridization of *BfHifα* with antisense probe and with sense probe. **(A-H)** From blastula to mid-neurula, *BfHifα* is ubiquitously expressed at a low level. **(I,J)** In two-day larvae, localized expression is found in the cerebral vesicle (arrow). **(K,L)** Cryosections of an amphioxus juvenile show *BfHifα* expression in the central nervous system (arrows), the pharyngeal bars (arrowheads), and the intestine (open arrowhead). The scale bar in A applies to A-J. Blastoporal views are labeled as ‘bp’, and panels E-J are lateral views; ‘d’ denotes the dorsal side of the larva in I. In E-H, anterior is to the left; in I and J, anterior is to the upper left.

The *BfHifα* was ubiquitously expressed at a very low level from blastula to mid-neurula stage (Figure 9A,C,E,G). With prolonged staining, tissue-specific expression was discovered in the cerebral vesicle (Figure 9I) during the larval stage. Cryosectioned samples of amphioxus juveniles showed that *BfHifα* was expressed in the CNS, the pharyngeal bars, and the intestine (Figure 9K).

## Discussion

### The *bHLH-PAS* genes of the *B. floridae* genome and the evolution of *bHLH-PAS* families in chordates

To discuss the *bHLH-PAS* genes, it is best to begin by reviewing the aliases of these bHLH-PAS families (orthologous gene clusters). First, the name ‘Bmal/cycle’ is used here for this family based on the naming used in previous reports [3,4,57], although McIntosh *et al*. suggested that mammalian *Arntl* (*Bmal1*) and *Arntl2* (*Bmal2*) be renamed as *Cycle1* and *Cycle2* based on their functions and expression patterns [7]. Second, for the NCOA/SRC family, we use NCOA as the family name as McIntosh *et al*. suggested [7], although some previous reports used SRC [3,4,57].

Before this study, amphioxus *bHLH-PAS* genes, including *Ncoa* of *B. belcheri*[58], *Hifα* of *B. belcheri tsingtauense* (*B. japonicum*) [59], *Bmal* of *B. lanceolatum*[60], and *Sim* of *B. floridae*[55], had been identified. The present study has confirmed the *Ncoa*, *Hifα*, and *Bmal* homologs of *B. floridae*, and identified six additional *bHLH-PAS* genes. Thus, we conclude that there are ten amphioxus *bHLH-PAS* genes in total, and nine of them correspond to nine well-known bHLH-PAS families shared by all bilaterians. The existence of nine amphioxus *bHLH-PAS* genes of conserved families is consistent with the previous suggestion that the number of these families is stable [4]. These nine bHLH-PAS families are shared by deuterostomes and protostomes, suggesting that they originated in the last common ancestor of all bilaterian animals. In vertebrates, many bHLH-PAS families have more than one paralog. For example, eight of nine human bHLH-PAS families have more than one member [4]. The emergence of multiple copies of these genes in vertebrates may be the result of vertebrate-specific whole-genome duplication and subsequent losses [28]. The vertebrate-specific duplicated genes may be subject to functional divergence by neofunctionalization or subfunctionalization [61].

The tenth amphioxus *bHLH-PAS* gene, *BfbHLHPAS-orphan*, was discovered in this study, and its putative ortholog in another amphioxus species (*B. belcheri*) was also identified by our BLAST search. Our phylogenetic analysis suggests that *BfbHLHPAS-orphan* may be related to arthropod *Met* genes and two spiralian predicted sequences (Figures 1 and 2). Extensive searches on various vertebrate genomes have not yet found an ortholog of *Met* or amphioxus ‘*bHLHPAS-orphans*’. It should be noted that Met proteins, which had been found only in arthropods [21], also contain bHLH, PAS A, and PAS B domains, but previous large-scale phylogenetic analyses on the bHLH superfamily had neglected them. Thus, Met proteins, BfbHLHPAS-orphan, and the two sequences (Ct199895 and Lg237855) from spiralians may make up another orthologous bHLH-PAS family, as we show in our phylogenetic analysis (Figures 1 and 2). It is possible that during chordate evolution the BfbHLHPAS-orphan has been retained in cephalochordates, but its ortholog was lost in vertebrates. Another possibility is that this gene family emerged independently in the amphioxus lineage and in protostomes by duplication or domain shuffling. Genome-wide analyses in more metazoan phyla for comparing the full complements of *bHLH-PAS* genes in their genomes should help to shed more light on this issue.

### Expression patterns of amphioxus *bHLH-PAS* genes shed light on the evolution of the bHLH-PAS superfamily

By comparing different animal models, similarities and differences of expression patterns of *bHLH-PAS* genes can be used to deduce the evolutionary themes of each *bHLH-PAS* family. Some amphioxus *bHLH-PAS* genes are expressed in patterns similar to those of their vertebrate homologs. This implies that amphioxus and vertebrates have comparable regulatory networks controlling these genes and that these networks may have origins in the common chordate ancestor over 520 million years ago. An example of conserved function was described for *Hifα* of another amphioxus species, *B. belcheri tsingtauense* (*B. japonicum*), with functions of oxygen-sensing, nuclear localization, and transcriptional regulation [59]. Although having conserved bHLH and PAS domains may imply functional stability by DNA-binding and dimerization, more biochemical evidence is required to properly elucidate the nature of amphioxus bHLH-PAS family members. Some amphioxus *bHLH-PAS* genes show different spatial expression patterns than those of their vertebrate homologs, suggesting changes in gene regulation after the divergence of the two lineages. The details of each family are discussed below.

#### The ARNT family

In amphioxus, *BfArnt* is expressed at two levels: first, it is broadly expressed at a low level; second, a higher level of expression specifically localizes in neural tissues. Previous studies indicate that many ARNT family members are broadly expressed; they act as a general dimerization partner that can heterodimerize with many bHLH-PAS proteins and activate or repress different sets of downstream genes [5,7]. Their function depends on their dimerization partners, and the existence of dimerization partners may be restricted by developmental spatial cues (*sim*, *trh*, *dys* in fly), by ligand-induced activity (vertebrate AHRs), or by hypoxia-dependent stability or activity (HIF family) [5,25,62-64]. Therefore, the basal and widespread expression of *BfArnt* may be consistent with other ARNT orthologs: a broadly expressed bHLH-PAS protein dimerization partner.

By contrast, the CNS-specific expression of *BfArnt* may be comparable to *Arnt2* in mice. Two murine ARNT paralogs have different expression patterns: *Arnt* is widely expressed, while *Arnt2* expression is more restricted to the neural-epithelium [62,65]. It is possible that the functions of the ancient *Arnt* gene were partitioned in vertebrate ARNT paralogs after the gene-duplication event [61].

#### The NCOA family

Our result shows that *BfNcoa* has CNS-specific expression. This may be comparable to vertebrate models. In the developing mouse embryo, *Ncoa1* (*SRC-1*) is highly expressed in olfactory epithelium, brain, anterior pituitary, and other organs [66,67]; mouse *Ncoa2* (*SRC-2*) is expressed in the developing anterior pituitary [68]. Similarly, *Xenopus* NCOA paralogs are expressed in various parts of the CNS [58]. These findings suggest that these vertebrate NCOA paralogs contribute to CNS development. In a previous study using the Asian amphioxus *B. belcheri*, *Ncoa* expression was not detected in the CNS, and it was proposed that NCOA expression may have shifted from non-CNS to CNS only in the vertebrate lineage (supplementary figure 4 in [58]). By contrast, our results clearly show that *BfNcoa* is indeed expressed in CNS during *B. floridae* embryogenesis. The difference between our results and those of Chen *et al*. [58] may stem from differences in species, experimental protocols, riboprobe sensitivity, or the developmental stage examined. In any event, our results suggest that *NCOA* function in the CNS is likely conserved in chordates. However, the NCOA homolog of fruit fly, *taiman*, is required in cell motility of ovarian follicular border cells and in axon migration [69,70], and little is known about whether NCOA homologs have a role in the CNS of non-chordates.

#### The AHR family

In well-studied animal models, the AHR family members have diverse functions. In fruit fly, *spineless* (the AHR homolog) is expressed in precursors of antenna, legs, and bristle, and it is required for normal development of these structures [71,72]. In *Caenorhabditis elegans*, *ahr-1* participates in specification of GABAergic neurons [73]. The vertebrate AHR family is comprised of AHR1, AHR2, and AHR repressor [74]. Vertebrate AHRs are required for the normal development of various organs, including nervous system and vascular system [75,76]. However, the well-known role of mammalian AHRs and AHR repressors is in the response to exposure to aromatic hydrocarbons, which was suggested to be a vertebrate innovation [74]. Mammalian AHRs (AHR1 and AHR2) are inducible by aryl hydrocarbons (including dioxin) and regulate the transcription of metabolic enzymes, while AHR repressors can repress the activity of AHRs (reviewed in [17,18]).

Amphioxus *BfAhr* is expressed in cells surrounding the mouth and in some cells in the epidermis of the rostrum. The former is reminiscent of *SoxB1c*-expressing ectodermal cells, which have been suggested to be neurogenic [77]; the latter is reminiscent of epidermal sensory neurons [78]. It is tempting to suggest that amphioxus *BfAhr*-expressing cells may be related to chemosensory neurons, and a neurogenic role of *BfAhr* is more like that in other protostomes. No clear *BfAhr* expression was discovered in the vascular system, so it is likely that the involvement of AHRs in vertebrate vascular development is a more recently derived characteristic.

#### The Sim family

*Sim* in fruit fly is expressed in ventral-lateral ectodermal cells and is required for CNS midline specification [5,79]. In mouse, the two Sim paralogs (*Sim1* and *Sim2*) are transcriptional repressors [80]. They are expressed in slightly different patterns: in the CNS, both are expressed in diencephalon, and *Sim1* expression extends caudally to the mesencephalon (midbrain); outside the CNS, the two paralogs are also expressed in different patterns [81]. Mouse *Sim1* is required for the normal development of the paraventricular nucleus and supraoptic nucleus in the hypothalamus [82], while mouse *Sim2* is required in the normal development of the palate, where no *Sim1* is expressed [83].

The expression of amphioxus *BfSim* in the anterior CNS and mesoendoderm has been described previously, and it was suggested that amphioxus *BfSim* expression marks the amphioxus homolog of the posterior diencephalon and midbrain [55]. Based on co-expression with *AmphiHu/Elav*, the six newly discovered cell clusters in the trunk CNS with *BfSim* expression (open arrowheads in Figure 7) in this study are likely to be postmitotic neurons. In addition, *BfSim* expression co-localizes with *BfArnt*. This suggests that the formation of a heterodimer for regulating downstream genes is a conserved function of these two factors [64,84].

#### The NPAS4/dysfusion family

Members of the NPAS4/dysfusion family have different functions in flies and mammals. In fruit fly, dysfusion dimerizes with tango (tgo, the fly ARNT homolog) and is required for the branching and fusion of tracheal cells [25,26]. In mammals, Npas4 dimerizes with Arnt2 or Arnt [85]. *Npas4* in mouse is expressed in the postnatal hippocampus [22] and *Npas4* in rat is required in the formation and retention of fear conditioning [24], but newborn *Npas4*^-/-^-mutant mice were morphologically indistinguishable from wild-types [23]. The expression pattern of amphioxus *BfNpas4* differed markedly from that of fruit fly or mammal; no expression was found in amphioxus embryonic CNS. It is possible that NPAS4/dysfusion members in these three lineages are regulated by different mechanisms.

#### The HIF family

Members of the HIF family participate in the hypoxia response in various animals. The stability and activity of HIF proteins are regulated by oxygen-dependent enzymes, and this mechanism is likely present in all animals [10]. ‘Invertebrate animals,’ from placozoa to amphioxus, have only one HIF member in their genomes, whereas mammals have three members of the HIF family: *Hif1α*, *Hif2α*, and *Hif3α*[10,86]. Three paralogs of mammalian HIF, with different functions, are retained in mammalian genomes. The functional differences between *Hif1α* and *Hif2α* may be the result of partitioning ancestral functions. However, the mammalian Hif3α protein is a transcriptional repressor, which is most likely a novel function that emerged in vertebrates. Under hypoxia, invertebrate HIFs or mammalian Hif1α or Hif2α proteins dimerize with ARNT members and activate downstream genes [15,16]. HIFs are also required in mammalian development, and the Hif1a^-/-^mouse is not viable and has CNS defects [87]. *Hif1α* mutations also impair the development of placenta, heart, and bones (reviewed in [86]).

Our results on the embryonic expression pattern of *BfHifα* are reminiscent of the pattern of *BfArnt*: a broad expression at low levels and stronger expression specifically localized to the CNS. These suggest two roles of *BfHifα*: first, the ubiquitous weak expression supports a function as a hypoxia sensor at the cellular level; and, second, the embryonic CNS-specific strong expression implies that it is required in normal neuronal development. The biochemical properties of another amphioxus species’ HIF protein have previously been characterized [59]. Similar to the previous report [59], we discovered different transcript isoforms of *BfHifα* in *B. floridae*. Isoforms that lack part of the oxygen-dependent degradation domain may be hydroxylated and then degraded under a slightly different oxygen level, providing a different level of regulation.

#### The ‘clock genes’ and circadian rhythm

*Bmal* and *Period* genes show expression oscillation in a bent dumbbell-shaped region in the cerebral vesicle of amphioxus [54,60]. Using Q-PCR to quantify mRNA, although we observed different *BfPeriod* expression levels between the day and night period, we could not detect significant differences in the expression levels of *BfClock* and *BfBmal* at different time points during the daylight cycle. For *Clock*, despite the fact that fly *dClock* has an oscillatory expression in fly heads [88], the murine *Clock* (*mClock*) mRNA and mClock protein show no diurnal oscillation in mouse brain [89,90]. For *Bmal*, the disparity between our Q-PCR result and *in situ* hybridization in the previous report may be due to different quantification methods. Semi-quantification by *in situ* hybridization and image processing may be more sensitive in locating expression changes in particular cell groups. Our Q-PCR result may be affected by other *BfBmal*-expressing cells - a previous study on laboratory rats reported that different nervous nuclei express clock-related genes with a dramatic antiphase [91].

### Comparison of amphioxus *bHLH-PAS* cDNA with current genomic scaffolds and gene models reveals limitations of the current *B. floridae* gene models

**Figure 10:**
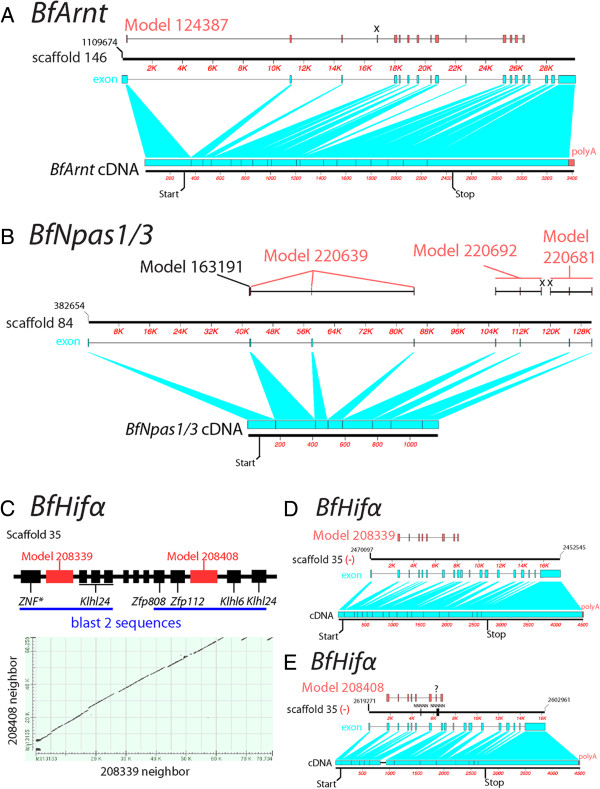
**Representative comparisons of obtained cDNA, amphioxus genomic DNA, and gene models.** The comparison of obtained cDNA with amphioxus genomic DNA scaffolds and gene models reveals problems with the gene models. In panels A, B, D, and E, the upper black band with coordinates represents part of the genomic scaffold. Red segmented boxes above the genomic scaffolds represent gene models. The lower black band represents cDNA. Exons are shown as cyan segmented boxes. Predicted exons not present in cDNA are labeled with ‘x’. Predicted exons with no evidence of existence are labeled with ‘?’. **(A)** The comparison of *BfArnt* cDNA, the corresponding genomic scaffold, and gene model 124387. This shows that, based on the model, the predicted structure of exon/intron is incorrect, and the model does not involve a putative translation stop. **(B)** The comparison of *BfNpas1/3* cDNA, the corresponding genomic scaffold, and gene models. This shows that those models lack the correct translation start and that four separate models should be combined to represent a single gene. **(C-E)** The comparison of *BfHifα* cDNA and the two corresponding genomic regions shows the redundancy of models. In panel C, the upper schematic figure (not to scale) shows the positional relationships of two *BfHifα* models (red boxes) and their neighboring gene models (black boxes), and the X-Y plot shows the comparisons of two scaffold regions denoted by blue lines. Synteny of gene models on each scaffold region and sequence similarity show the redundancy of the gene models. Panels D and E show the comparison of *BfHifα* cDNA (long isoform), the genomic scaffolds, and gene models 208339 and 208408. In E, the black boxes on the genomic scaffold are ambiguous gap regions, which were not sequenced and were denoted as strings of ‘N’s in the genome browser. The cDNA region not aligned may be due to these regions.

In this study, we also used experimentally verified cDNA sequences of amphioxus *bHLH-PAS* genes to assess the quality of the current amphioxus gene models. We mapped the exon-intron structures of transcript models on the JGI website onto the genomic scaffolds and compared them to our cDNA sequences. As the comparison shows, most presumed exons were correctly predicted; however, many differences between the models and the obtained cDNAs were discovered (Figure 10 and Additional file 7: Figure S5). We summarize here four major types of discrepancies between the existing gene model set and our cDNA sequences: First, inaccurate exon/intron structures were presented in certain gene models, including *BfNcoa*, *BfArnt*, *BfHifα*, *BfbHLHPAS-orphan*, *BfClock*, *BfBmal*, *BfAhr*, and *BfNpas1/3* (Figure 10A and Additional file 7: Figure S5A-O,R-T). Second, translation start and/or stop sites were incorrectly predicted for *BfNcoa*, *BfArnt*, *BfHifα*, *BfbHLHPAS-orphan*, *BfClock*, and *BfNpas1/3* (Figure 10B and Additional file 7: Figure S5A-K,R-T). Third, multiple gene models should be joined to represent a single gene. This was the case for *BfAhr* and *BfNpas1/3* (Figure 10B; Additional file 7: Figure S5O,S). Fourth, redundancy of models: In the cases of *BfHifα*, *BfbHLHPAS-orphan*, *BfClock*, *BfBmal*, and *BfNpas1/3*, two genomic scaffolds or two regions of the same scaffold were hit in the searches (Figure 10C-E and Additional file 7: Figure S5C-N,R-T).

To our knowledge, our study is likely the first attempt to comprehensively annotate an amphioxus gene family using both computer-predicted gene models and experimentally verified cDNA sequences. Due to the high genetic variation between the two haplotypes of the *B. floridae* genome, it has been reported that the two alleles of a single locus are frequently represented by separate gene models in the current assembly [28,92]. By careful comparisons, we have been able to extract the most representative gene model for each *bHLH-PAS* family gene in *B. floridae*. However, we found that eight out of the ten *B. floridae bHLH-PAS* genes are depicted by problematic gene models in the current genome assembly. Our discovery calls for more attention to the current *B. floridae* genome assembly and gene model annotation. Because the cephalochordate amphioxus is widely considered as a key organism for understanding the evolution of chordates [93], information about its genome, especially the protein-coding gene contents, are frequently used in comparative genomic analyses [94,95]. Our results show that the existing set of *B. floridae* gene models may contain many problematic models. To improve the current amphioxus gene model annotation, more data from experimentally verified transcripts will need to be incorporated into gene model prediction. With the advance of the high-throughput next-generation sequencing technologies, we anticipate that next-generation sequencing transcriptome data from RNA-sequencing analysis will help to address this issue.

## Conclusions

In this study, we identified ten *bHLH-PAS* genes from the amphioxus genome and determined the embryonic expression profiles for these genes. In addition to the nine currently recognized *bHLH-PAS* families, our survey across various bilaterian genomes suggests that the tenth amphioxus *bHLH-PAS* member (*BfbHLHPAS-orphan*) along with arthropod *Met* genes and the two newly identified spiralian bHLH-PAS-containing sequences may represent an ancient group of genes that was already present in the common ancestor of bilaterian animals but lost in the vertebrate lineage. Our expression analysis using *in situ* hybridization not only provides new spatial expression information on three previously unknown genes - *Arnt*, *Ahr*, and *Npas4* - and on *Hifα*, but also provides clear evidence to revise previous descriptions of the embryonic expression of amphioxus *Ncoa* and *Sim* genes. Thus, our results provide a more accurate account for further comparative studies. Comparing the expression patterns of the vertebrate *bHLH-PAS* paralogs, which are the result of whole-genome duplication, we found that although several members seem to retain conserved expression patterns during chordate evolution, many duplicated paralogs may have undergone subfunctionalization and neofunctionalization in the vertebrate lineage. The discovery that *Arnt*, *Ncoa*, *Sim*, and *Hifα* are expressed in certain domains within the developing CNS in both amphioxus and vertebrates suggests the functional conservation of these genes in chordate CNS development. Moreover, we found that *Arnt* and *Sim* are co-expressed in six post-mitotic neuronal cell clusters within the amphioxus CNS, which is consistent with their functions in forming heterodimers to regulate downstream targets in model vertebrates. Further characterization of these specific neuronal cell clusters in amphioxus CNS and their comparison to vertebrate CNS neurons may provide more information on the organization and evolution of CNS neurons in chordates.

## Competing interests

The authors declare that they have no competing interests.

## Authors’ contributions

KLL and JKY designed the study. KLL carried out gene orthology analyses, PCR cloning, sequence alignment, phylogenetic analyses, *in situ* hybridization, gene expression analyses, and imaging. TML contributed to *BfHifα in situ* hybridization, gene expression analyses, and imaging. KLL and JKY wrote the manuscript. All authors read and approved the final manuscript.

## Supplementary Material

Additional file 1: Table S1 List of PCR primers used for amplifying cDNA fragments of *B. floridae bHLH-PAS* genes. Click here for file

Additional file 2: Table S2 List of Q-PCR primers. Click here for file

Additional file 3: Figure S1 Distribution of conserved domains of amphioxus and representative human bHLH-PAS proteins. Schematic diagrams, drawn approximately to scale, showing conserved domains of representative human (Hs, black bars) and amphioxus (Bf, yellow bars) bHLH-PAS proteins. All of the amphioxus bHLH-PAS proteins have conserved bHLH, PAS A, and PAS B domains. A further comparison is made between the well-characterized human HIF1α and the BfHifα proteins: presumed oxygen-dependent degradation domain (ODDD), C-terminal trans-activation domain (CTAD), and hydroxylation target residues of BfHifα proteins are labeled to show their structural similarity. The short isoform of BfHifα (s) lacks the N-terminal part of presumed ODDD, including one presumed hydroxylation target proline. The human proteins used were the same as those used in database searching. Click here for file

Additional file 4: Figure S2 Alignments of conserved domains of representative human (Hs) and amphioxus (Bf) bHLH-PAS proteins. Positions with high similarity (under BLOSUM62 matrix) shared by over 70% of sequences are color-shaded. The long isoform of BfHifα protein, ‘Bf_Hifa(L),’ is shown. The BfbHLHPAS-orphan is labeled as ‘Bf_orphan.’ **(A)** Alignment of the bHLH domain. Designation of basic, Helix 1, Loop, and Helix 2 regions is based on Ferre-D’Amare *et al*. [1]. **(B)** Alignment of the PAS A domain. **(C)** Alignment of the PAS B domain. For amphioxus BfAhr and BfNpas1/3 proteins, the predicted protein sequences from cDNA fragments only contain partial PAS B domain. Click here for file

Additional file 5: Figure S3 Sequence alignments showing presumed conserved hydroxylation sites of HIF homologs. Sequences of HIF homologs are aligned, and the presumed hydroxylation sites are highlighted by red boxes. The proteins analyzed all have comparable hydroxylation targets, except the short isoform of BfHifα. The following proteins are used: Bf, *Branchiostoma floridae*, this study; Hs, *Homo sapiens*, Q16665.1; Mm, *Mus musculus*, NP_034561.2; Xl, *Xenopus laevis* (African clawed frog), NP_001080449.1; Dr, *Danio rerio* (zebra fish), AAQ91619.1; Sp, *Strongylocentrotus purpuratus* (sea urchin), an unpublished sequence from Dr. Yi-Hsien Su’s laboratory; Tc, *Tribolium castaneum* (red flour beetle), XP_967427.2; Pp, *Palaemonetes pugio* (grass shrimp), AAT72404. Click here for file

Additional file 6: Figure S4 Quantification of circadian rhythm related genes. Q-PCR results showed the expression levels of ‘clock genes’ in amphioxus juveniles’ anterior part, including their cerebral vesicle. Error bars show the standard deviation of three biological replicates. The expression levels of *BfClock* and *BfBmal* show no significant difference between two sample groups (light-phase versus dark-phase). However, the expression level of *BfPeriod* in light-phase group is significantly higher (t-test: *P* <0.05) than that in dark-phase group. Click here for file

Additional file 7: Figure S5 Relationships of obtained cDNA, *B. floridae* genomic scaffolds, and gene models of *bHLH-PAS* genes. For all *bHLH-PAS* genes of *B. floridae*, we mapped the exon-intron structures of transcript models from the JGI database onto the genomic scaffolds and compared them to the cDNA sequences we obtained. Panels A, B, D, E, G, H, J, K, M-Q, S, and T show comparison of obtained cDNA, genomic scaffolds, and corresponding gene models; panels C, F, I, L, and R show the comparisons of redundant models and neighboring genomic regions. Detailed descriptions are included at the end of the figure. Click here for file
