# Supplementary figures and images for: Genome-wide survey and expression analysis of the bHLH-PAS genes in the amphioxus Branchiostoma floridae reveal both conserved and diverged expression patterns between cephalochordates and vertebrates
Source: EvoDevo. 2014 Jun 3;5:20. doi: 10.1186/2041-9139-5-20 (PMC4066832; doi:10.1186/2041-9139-5-20)

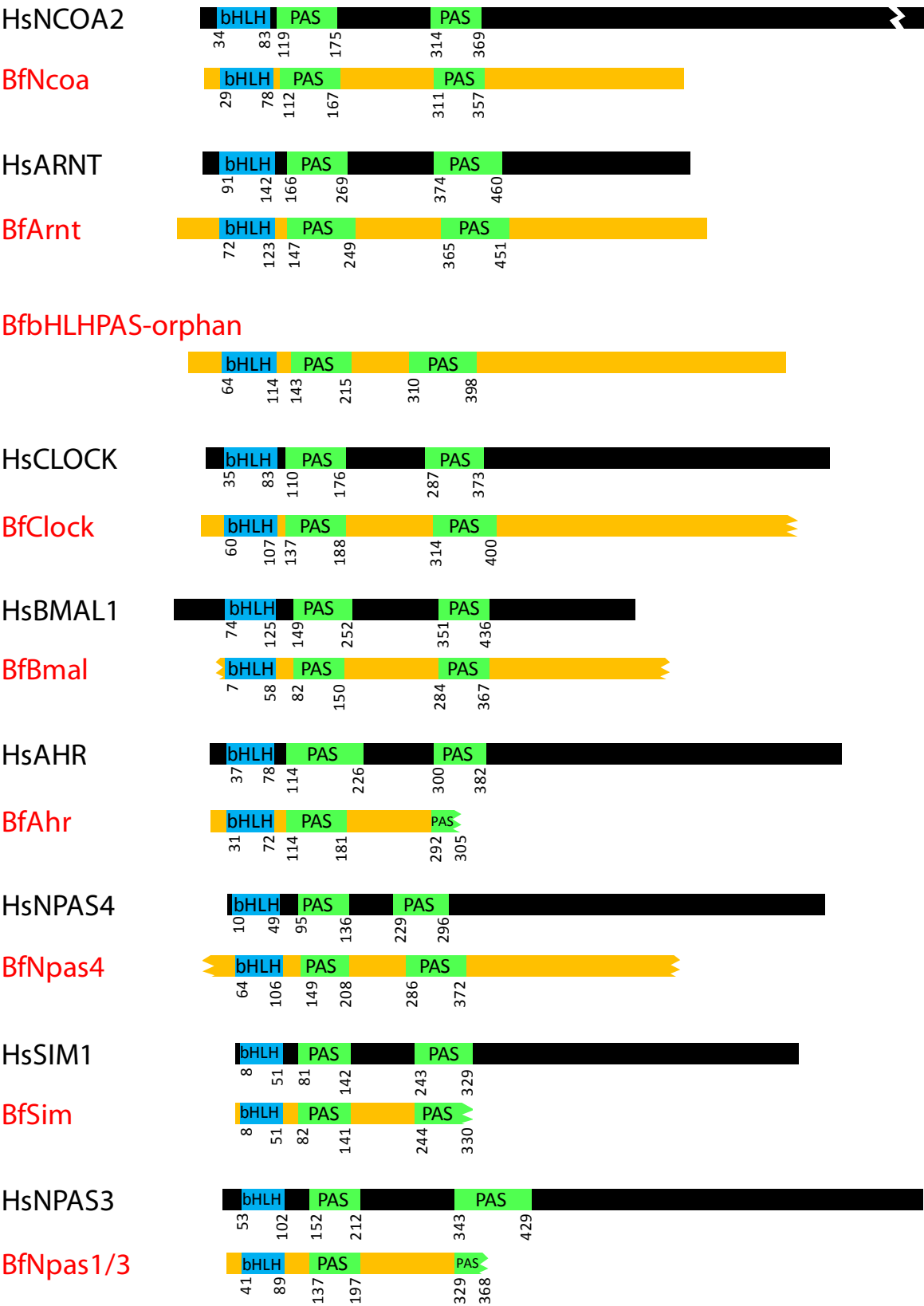

HsHIF1α

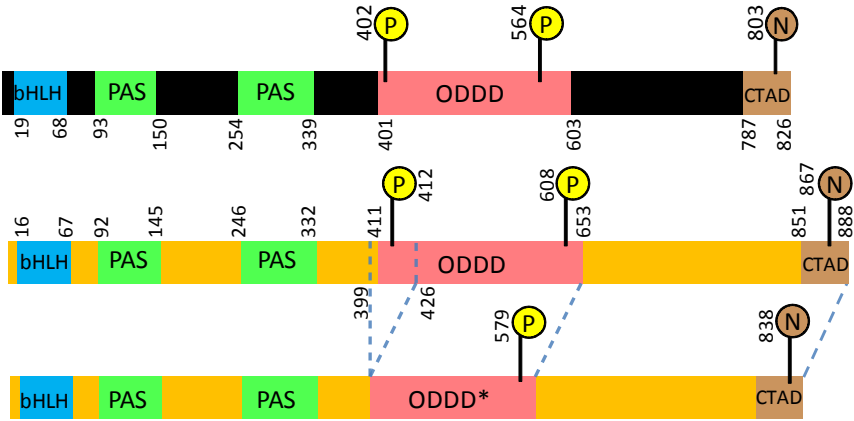

BfHifa (L)

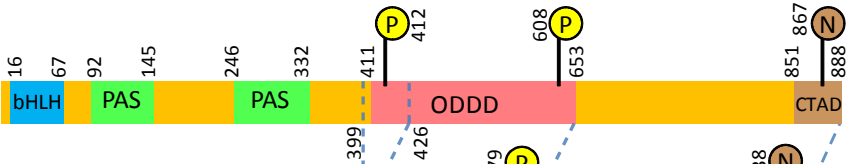

BfHifa (s)

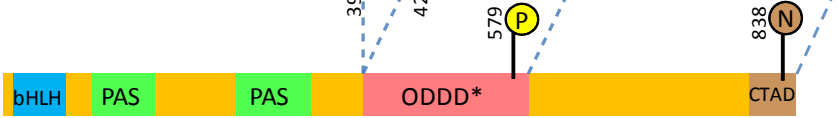

Supplement: Additional file 3: Figure S1 — Distribution of conserved domains of amphioxus and representative human bHLH-PAS proteins. Schematic diagrams, drawn approximately to scale, showing conserved domains of representative human (Hs, black bars) and amphioxus (Bf, yellow bars) bHLH-PAS proteins. All of the amphioxus bHLH-PAS proteins have conserved bHLH, PAS A, and PAS B domains. A further comparison is made between the well-characterized human HIF1α and the BfHifα proteins: presumed oxygen-dependent degradation domain (ODDD), C-terminal trans-activation domain (CTAD), and hydroxylation target residues of BfHifα proteins are labeled to show their structural similarity. The short isoform of BfHifα (s) lacks the N-terminal part of presumed ODDD, including one presumed hydroxylation target proline. The human proteins used were the same as those used in database searching. [file 2041-9139-5-20-S3.pdf]

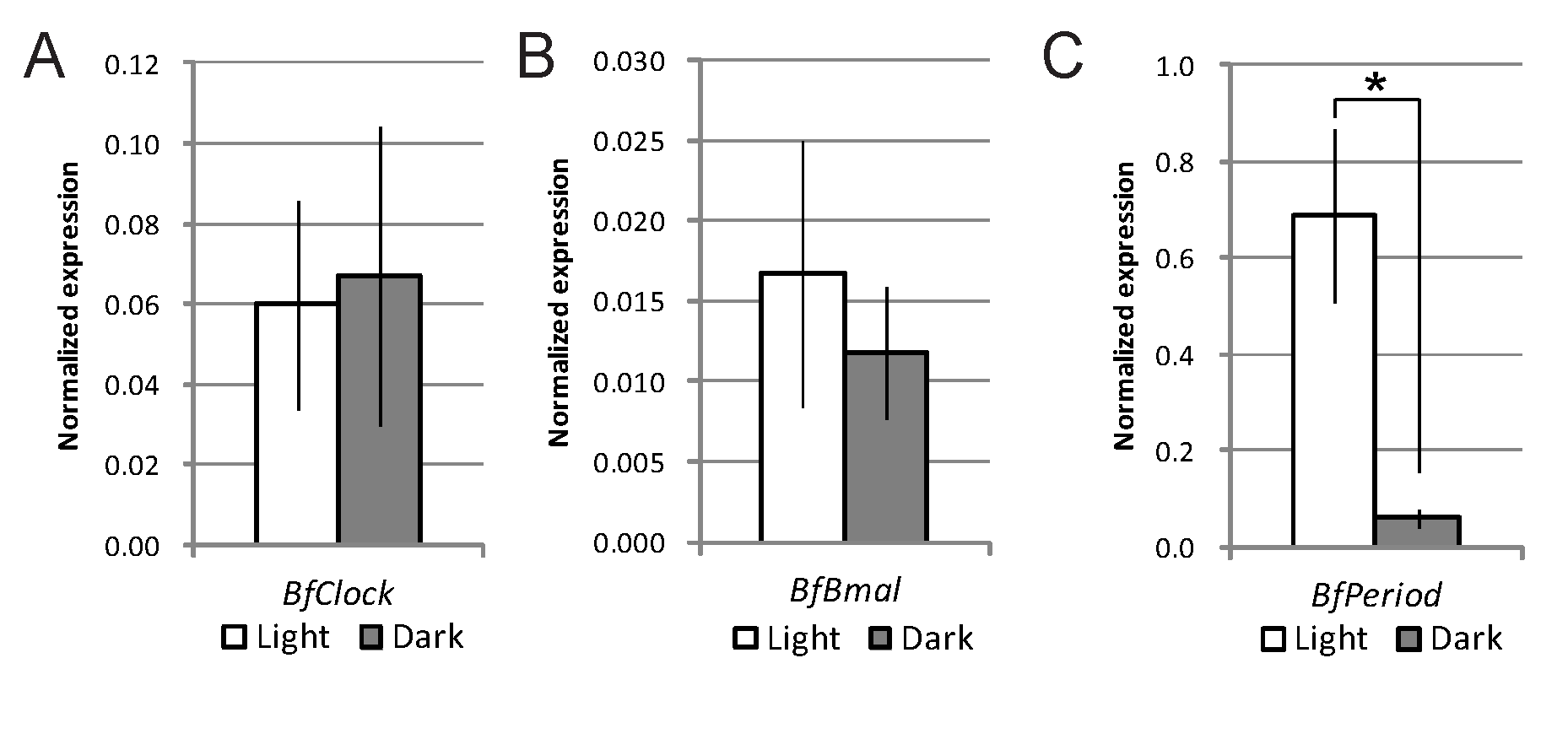

Supplement: Additional file 6: Figure S4 — Quantification of circadian rhythm related genes. Q-PCR results showed the expression levels of ‘clock genes’ in amphioxus juveniles’ anterior part, including their cerebral vesicle. Error bars show the standard deviation of three biological replicates. The expression levels of BfClock and BfBmal show no significant difference between two sample groups (light-phase versus dark-phase). However, the expression level of BfPeriod in light-phase group is significantly higher (t-test: P <0.05) than that in dark-phase group. [file 2041-9139-5-20-S6.tiff]
