# Supplementary material for: Genome-wide survey and expression analysis of the bHLH-PAS genes in the amphioxus Branchiostoma floridae reveal both conserved and diverged expression patterns between cephalochordates and vertebrates
Source: EvoDevo. 2014 Jun 3;5:20. doi: 10.1186/2041-9139-5-20 (PMC4066832; doi:10.1186/2041-9139-5-20)
Supplement: Additional file 4: Figure S2 — Alignments of conserved domains of representative human (Hs) and amphioxus (Bf) bHLH-PAS proteins. Positions with high similarity (under BLOSUM62 matrix) shared by over 70% of sequences are color-shaded. The long isoform of BfHifα protein, ‘Bf_Hifa(L),’ is shown. The BfbHLHPAS-orphan is labeled as ‘Bf_orphan.’ (A) Alignment of the bHLH domain. Designation of basic, Helix 1, Loop, and Helix 2 regions is based on Ferre-D’Amare et al. [1]. (B) Alignment of the PAS A domain. (C) Alignment of the PAS B domain. For amphioxus BfAhr and BfNpas1/3 proteins, the predicted protein sequences from cDNA fragments only contain partial PAS B domain. [file 2041-9139-5-20-S4.pdf]

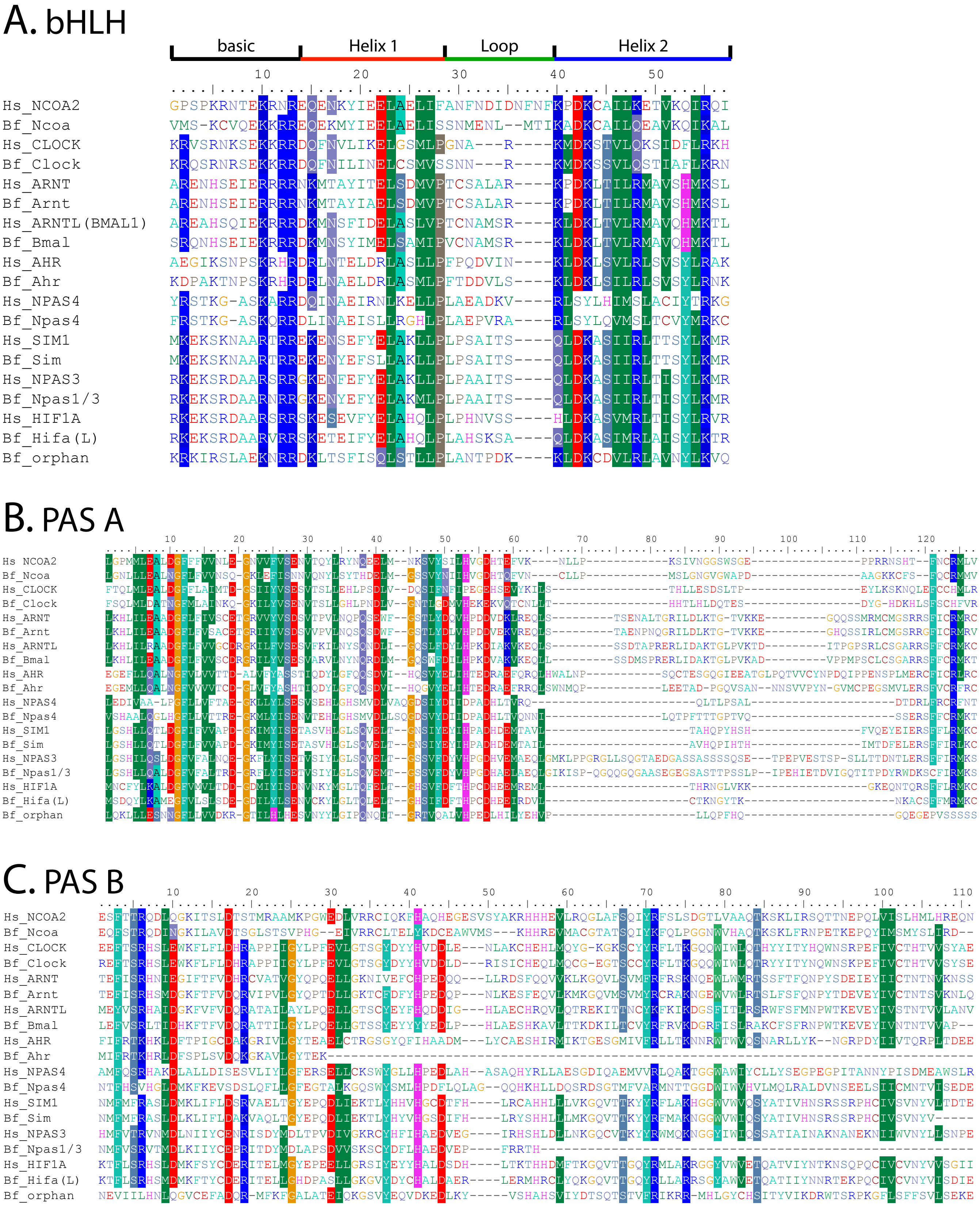
A. bHLH
basic
Helix 1
Loop
Helix 2
10 20 30 40 50
Hs_NCOA2 GPSPKRNTEKRNREQENKYIEELAELIFANFNDIDNFNFKPDKCAILKETVKQIRQI
Bf_Ncoa VMS-KCVQEKKRREQEKMYIEELAELISSNMENL--MTIKADKCAILQEAVKQIKAL
Hs_CLOCK KRVSRNKSEKKRRDQFNVLIKELGSMLPGNA---R----KMDKSTVLQKSIDFLRKH
Bf_Clock KRQSRNRSEKKRRDQFNILINELCSMVSSNN---R----KMDKSSVLQSTIAFLKRN
Hs_ARNT ARENHSEIERRRRNKMTAYITELSDMVPTCSALAR----KPDKLTILRMAVSHMKSL
Bf_Arnt ARENHSEIERRRRNKMTAYIAELSDMVPTCSALAR----KPDKLTILRMAVSHMKSL
Hs_ARNTL(BMAL1) AREAHSQIEKRRRDKMNSFIDELASLVPTCNAMSR----KLDKLTVLRMAVQHMKTL
Bf_Bmal SRQNHSEIEKRRRDKMNSYIMELSAMIPVCNAMSR----KLDKLTVLRMAVQHMKTL
Hs_AHR AEGIKSNPSKRHRDRLNTELDRLASLLPFPQDVIN----KLDKLSVLRLSVSYLRAK
Bf_Ahr KDPAKTNPSKRHRDRLNAELDRLASMLPFTDDVLS----KLDKLSILRLSVSYLRNK
Hs_NPAS4 YRSTKG-ASKARRDQINAEIRNLKELLPLAEADKV----RLSYLHIMSLACIYTRKG
Bf_Npas4 FRSTKG-ASKQRRDLINAEISLLRGHLPLAEPVRA----RLSYLQVMSLTCVYMRKC
Hs_SIM1 MKEKSKNAARTRREKENSEFYELAKLLPLPSAITS----QLDKASIIRLTTSYLKMR
Bf_Sim MKEKSKNAARTRREKENYEFSLLAKLLPLPSAITS----QLDKASIIRLTTSYLKMR
Hs_NPAS3 RKEKSRDAARSRRGKENFEFYELAKLLPLPAAITS----QLDKASIIRLTISYLKMR
Bf_Npas1/3 RKEKSRDAARNRRGKENYEFYELAKMLPLPAAITS----QLDKASIIRLTISYLKMR
Hs_HIF1A RKEKSRDAARSRRSKESEVFYELAHQLPLPHNVSS----HLDKASVMRLTISYLRVR
Bf_Hifa(L) RKEKSRDAARVRRSKETEIFYELAHQLPLAHSKSA----QLDKASIMRLAISYLKTR
Bf_orphan KRKIRSLAEKNRRDKLTSFISQLSTLLPLANTPDK----KLDKCDVLRLAVNYLKVQ
B. PAS A
10 20 30 40 50 60 70 80 90 100 110 120
Hs_NCOA2
Bf_Ncoa
Hs_CLOCK
Bf_Clock
Hs_ARNT
Bf_Arnt
Hs_ARNTL
Bf_Bmal
Hs_AHR
Bf_Ahr
Hs_NPAS4
Bf_Npas4
Hs_SIM1
Bf_Sim
Hs_NPAS3
Bf_Npas1/3
Hs_HIF1A
Bf_Hifa(L)
Bf_orphan
C. PAS B
10 20 30 40 50 60 70 80 90 100 110
Hs_NCOA2
Bf_Ncoa
Hs_CLOCK
Bf_Clock
Hs_ARNT
Bf_Arnt
Hs_ARNTL
Bf_Bmal
Hs_AHR
Bf_Ahr
Hs_NPAS4
Bf_Npas4
Hs_SIM1
Bf_Sim
Hs_NPAS3
Bf_Npas1/3
Hs_HIF1A
Bf_Hifa(L)
Bf_orphan
