# Supplementary material for: Genome-wide survey and expression analysis of the bHLH-PAS genes in the amphioxus Branchiostoma floridae reveal both conserved and diverged expression patterns between cephalochordates and vertebrates
Source: EvoDevo. 2014 Jun 3;5:20. doi: 10.1186/2041-9139-5-20 (PMC4066832; doi:10.1186/2041-9139-5-20)
Supplement: Additional file 5: Figure S3 — Sequence alignments showing presumed conserved hydroxylation sites of HIF homologs. Sequences of HIF homologs are aligned, and the presumed hydroxylation sites are highlighted by red boxes. The proteins analyzed all have comparable hydroxylation targets, except the short isoform of BfHifα. The following proteins are used: Bf, Branchiostoma floridae, this study; Hs, Homo sapiens, Q16665.1; Mm, Mus musculus, NP_034561.2; Xl, Xenopus laevis (African clawed frog), NP_001080449.1; Dr, Danio rerio (zebra fish), AAQ91619.1; Sp, Strongylocentrotus purpuratus (sea urchin), an unpublished sequence from Dr. Yi-Hsien Su’s laboratory; Tc, Tribolium castaneum (red flour beetle), XP_967427.2; Pp, Palaemonetes pugio (grass shrimp), AAT72404. [file 2041-9139-5-20-S5.pdf]

Proline hydroxylation site 1 in the ODDD

```
Bf Hifa(L)   GFFWDGEQALRDSPEDLTRVAPAAGDAMIPLGFPSADVLFGSSSLPDTS
Bf Hifa(s)   GFFWDGEQ----------------------------DVLFG-SSLPDTS
Hs HIF1A     SLFDK----LKKEPDALTLLAPAAGDTIISLDFGSNDTETDDQQLEEVP
Mm Hif1a     CLFDK----LKKEPDALTLLAPAAGDTIISLDFGSDDTETEDQQLEDVP
Xl Hif1a     CLFDK----LKQEPESLTVLAPDAGDEIIPLDFSSGD---SDKPYEDVP
Dr Hif1a     TLYNK----LKEEPEALTVLAPAAGDAIISLDFNNSDS--DIQLLKEVP
Sp Hif1      PLYLAGCNSSQAVEEKLAYLAPTAGDVMIELDPSDSQRMDLTSEFKEFN
Tc Hif1a     SDEEPGLTMLKDEPDDLTHLAPVAGDVCVPLDDHPFLTDKMLDDFLLRD
Pp Hif1a     PEDQPYGVELKDEPDDLTHLAPSGGDTCVPLEVPIFKPELDDVFTQI--
```

Proline hydroxylation site 2 in the ODDD

```
Bf Hifa(L)   PSLALDTGQPIQNNIRTKAAGKLTKRPLTAEELSYRAPYIPAYQMPLNPPNNAVLASLLETDRRQSVTSSRSY
Bf Hifa(s)   PSLALDTGQPIQNNIRTTAAGKLTKRPLTAEELSYRAPYIPAYQMPLNPPNNAVLASLLETDRRQSVTSSRSY
Hs HIF1A     KLFAEDT--EAKNPFSTQDT---------DLDLEMLAPYIPM-------DDDFQLRSFDQLSPLESSSASPES
Mm Hif1a     KLFAEDT--EAKNPFSTQDT---------DLDLEMLAPYIPM-------DDDFQLRSFDQLSPLESNSPSPPS
Xl Hif1a     KLFAIDT--EAKAPFTTQET---------DLDLEMLAPYIPM-------DDDFQLRTFDQLSSLECDSSIPQT
Dr Hif1a     KLFAIDT--EAKTPFSTQPM--------EDLDLEMLAPYIPM-------DDDFQLRIPSPLDPLPSATHSVSA
Sp Hif1      ------------------------------DELAMRAPYIPM-------GEDFDLSTDPLLGDMDPFTSETWM
Tc Hif1a     DSLSDED---RLSTFMNLHTE-------DESDLVAKAPYITMN-----MGDDLPLLMSDDLMWSSSEKSRASP
Pp Hif1a     NETAHESYVNIELKVENQNMD--------LDEFDMRAPFIPL-------SNELLMLNQDDLMWGAQPDSLPMG
```

The CTAD, highlighting asparagine hydroxylation site

```
Bf Hifa(L)   DLDDDF------LPPLTRADVEVNAPIQ-TYKLLQGNDLMSALDQSAALF
Bf Hifa(s)   ALDDDF------LPPLTRADVEVNAPIQ-TYKLLQGNDLMSALDQSAALF
Hs HIF1A     SMDESG------LPQLTSYDCEVNAPIQGSRNLLQGEELLRALDQVN---
Mm Hif1a     SMDESG------LPQLTSYDCEVNAPIQGSRNLLQGEELLRALDQVN---
Xl Hif1a     SFDGTV------LPQLTGYDCEVNAPVHGTRNLLQGEELLRALDQAN---
Dr Hif1a     SLEGSGG-----LPQLTRYDCEVNAPVQDRHHLLQGEELLRALDQVN---
Sp Hif       MPLGAV------LPLITNLDAEVNAPLPNTNRLLQGEDLLQALDLTAPY-
Tc Hif1a     LSLLDPDATIPSLLDLTQQDFEVNAPVN-S-LLLQGTDLLTALDINGPI-
Pp Hif1a     LLLVDPDLTIPSLSELSQLDFEVNAPAN-IGNLLQGADLLMALDQA----
```
