# Supplementary material for: Genome-wide survey and expression analysis of the bHLH-PAS genes in the amphioxus Branchiostoma floridae reveal both conserved and diverged expression patterns between cephalochordates and vertebrates
Source: EvoDevo. 2014 Jun 3;5:20. doi: 10.1186/2041-9139-5-20 (PMC4066832; doi:10.1186/2041-9139-5-20)
Supplement: Additional file 7: Figure S5 — Relationships of obtained cDNA, B. floridae genomic scaffolds, and gene models of bHLH-PAS genes. For all bHLH-PAS genes of B. floridae, we mapped the exon-intron structures of transcript models from the JGI database onto the genomic scaffolds and compared them to the cDNA sequences we obtained. Panels A, B, D, E, G, H, J, K, M-Q, S, and T show comparison of obtained cDNA, genomic scaffolds, and corresponding gene models; panels C, F, I, L, and R show the comparisons of redundant models and neighboring genomic regions. Detailed descriptions are included at the end of the figure. [file 2041-9139-5-20-S7.pdf]

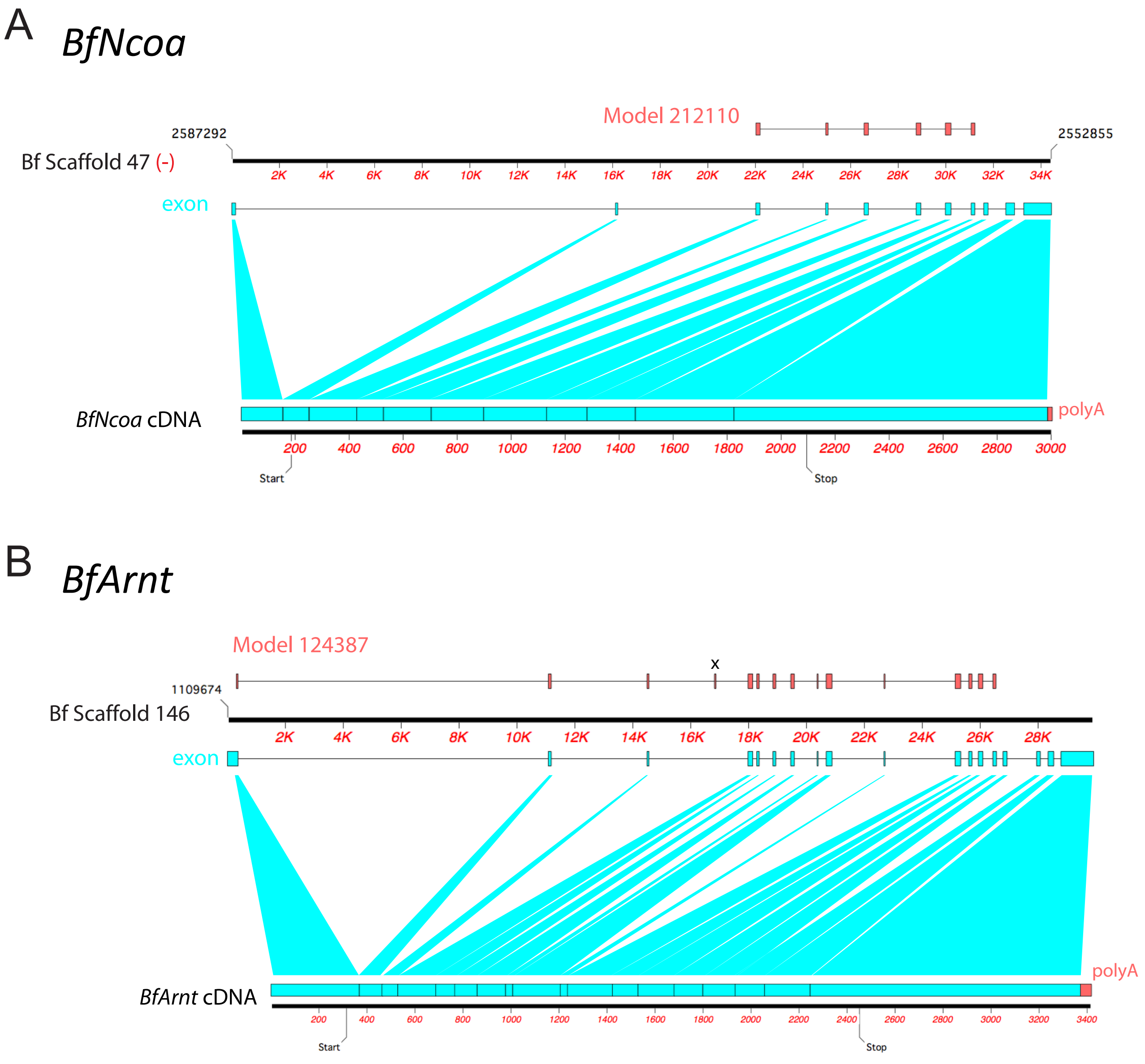
A
BfNcoa
Model 212110
2587292
2552855
Bf Scaffold 47 (-)
2K 4K 6K 8K 10K 12K 14K 16K 18K 20K 22K 24K 26K 28K 30K 32K 34K
exon
BfNcoa cDNA
polyA
200 400 600 800 1000 1200 1400 1600 1800 2000 2200 2400 2600 2800 3000
Start
Stop
B
BfArnt
Model 124387
x
1109674
Bf Scaffold 146
2K 4K 6K 8K 10K 12K 14K 16K 18K 20K 22K 24K 26K 28K
exon
BfArnt cDNA
polyA
200 400 600 800 1000 1200 1400 1600 1800 2000 2200 2400 2600 2800 3000 3200 3400
Start
Stop

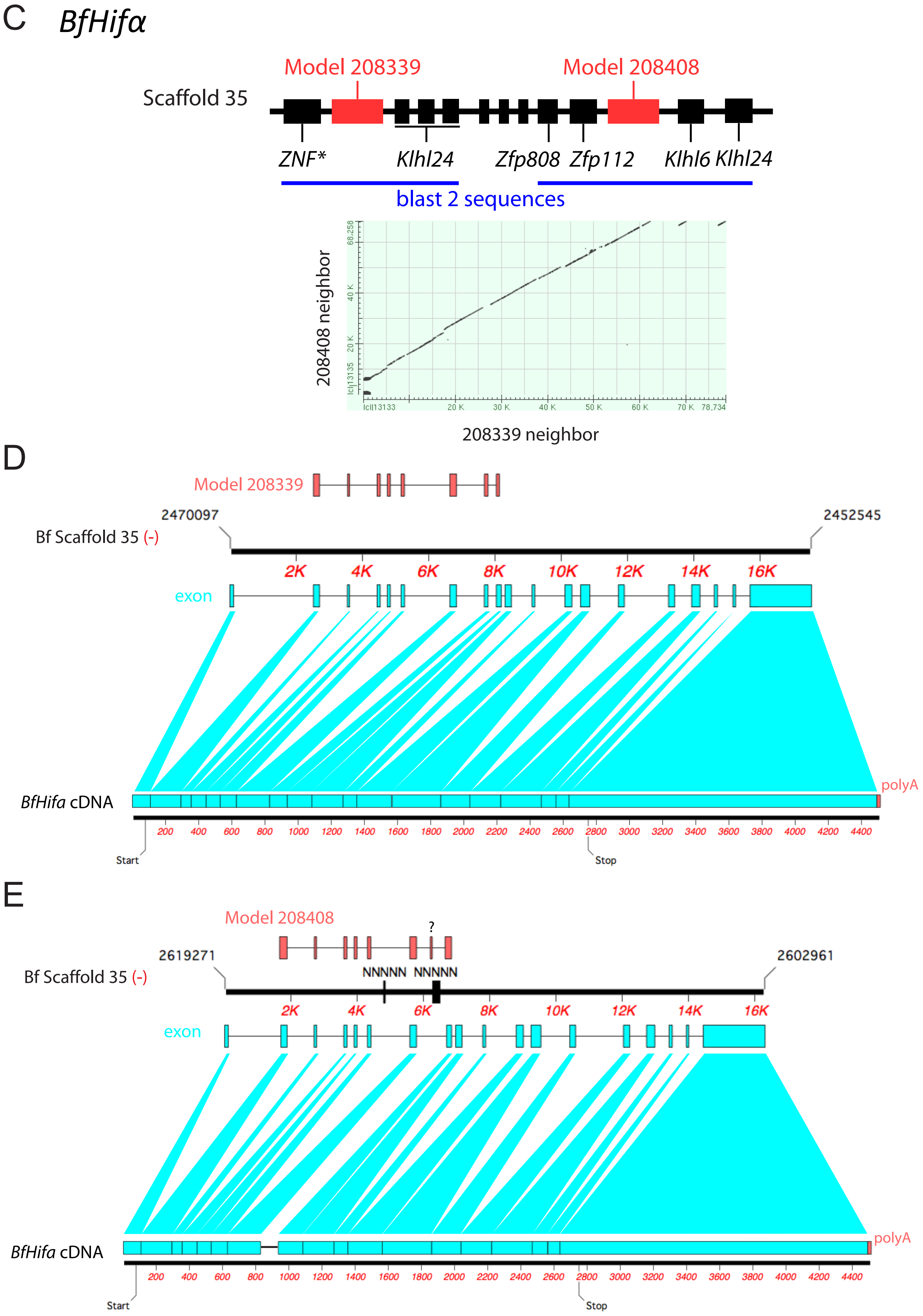
C
BfHifα
Model 208339
Model 208408
Scaffold 35
ZNF*
Klhl24
Zfp808
Zfp112
Klhl6
Klhl24
blast 2 sequences
208408 neighbor
68,258
40 K
20 K
lcl|13135
lcl|13133
20 K
30 K
40 K
50 K
60 K
70 K
78,734
208339 neighbor
D
Model 208339
2470097
2452545
Bf Scaffold 35 (-)
2K
4K
6K
8K
10K
12K
14K
16K
exon
BfHifa cDNA
polyA
200
400
600
800
1000
1200
1400
1600
1800
2000
2200
2400
2600
2800
3000
3200
3400
3600
3800
4000
4200
4400
Start
Stop
E
Model 208408
?
2619271
2602961
Bf Scaffold 35 (-)
NNNNN
NNNNN
2K
4K
6K
8K
10K
12K
14K
16K
exon
BfHifa cDNA
polyA
200
400
600
800
1000
1200
1400
1600
1800
2000
2200
2400
2600
2800
3000
3200
3400
3600
3800
4000
4200
4400
Start
Stop

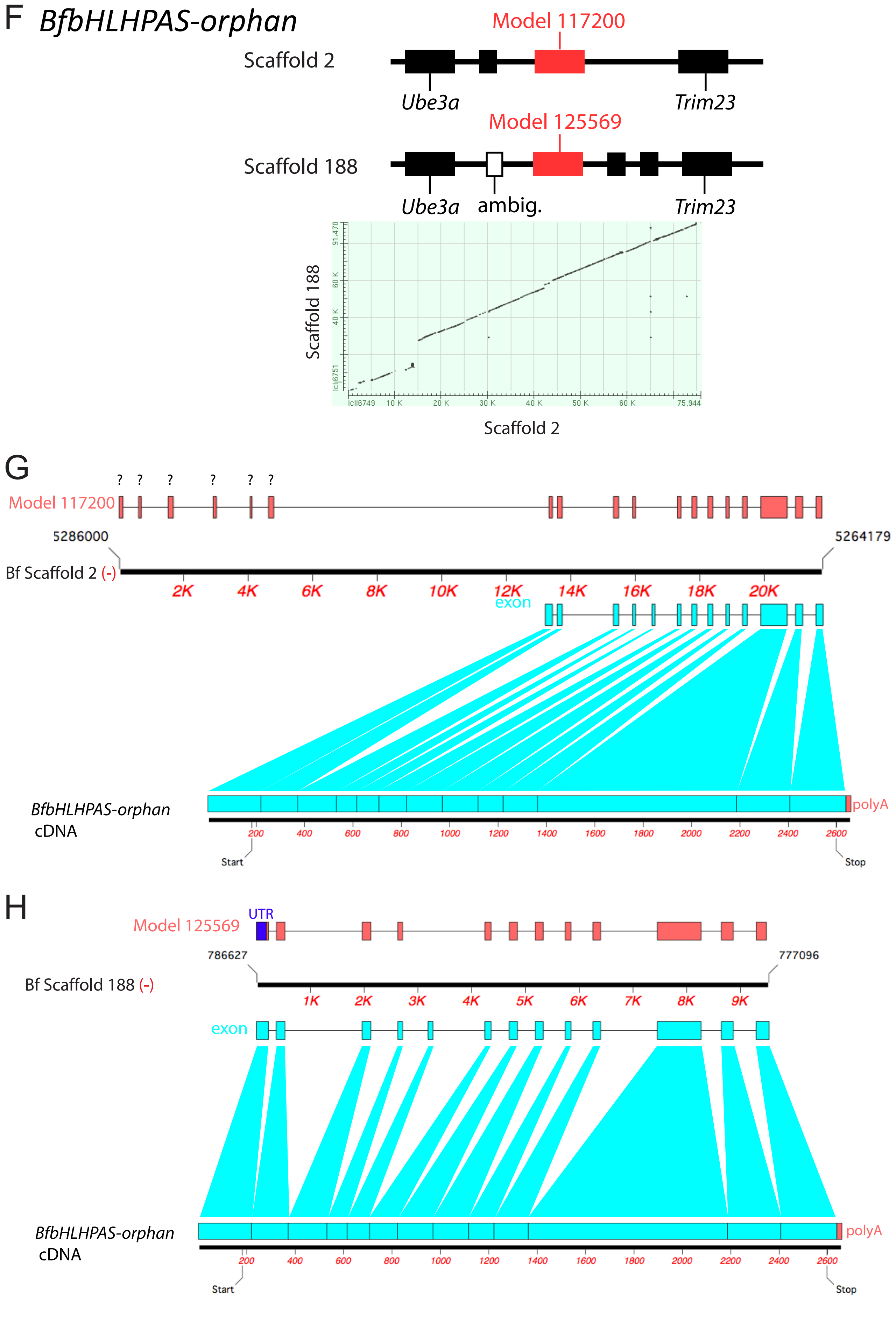
F
BfbHLHPAS-orphan
Model 117200
Scaffold 2
Ube3a
Trim23
Model 125569
Scaffold 188
Ube3a
ambig.
Trim23
Scaffold 188
91,470
60 K
40 K
lcl|6751
lcl|6749
10 K
20 K
30 K
40 K
50 K
60 K
75,944
Scaffold 2
G
? ? ? ? ? ?
Model 117200
5286000
5264179
Bf Scaffold 2 (-)
2K 4K 6K 8K 10K 12K 14K 16K 18K 20K
exon
BfbHLHPAS-orphan
cDNA
polyA
200 400 600 800 1000 1200 1400 1600 1800 2000 2200 2400 2600
Start
Stop
H
UTR
Model 125569
786627
777096
Bf Scaffold 188 (-)
1K 2K 3K 4K 5K 6K 7K 8K 9K
exon
BfbHLHPAS-orphan
cDNA
polyA
200 400 600 800 1000 1200 1400 1600 1800 2000 2200 2400 2600
Start
Stop

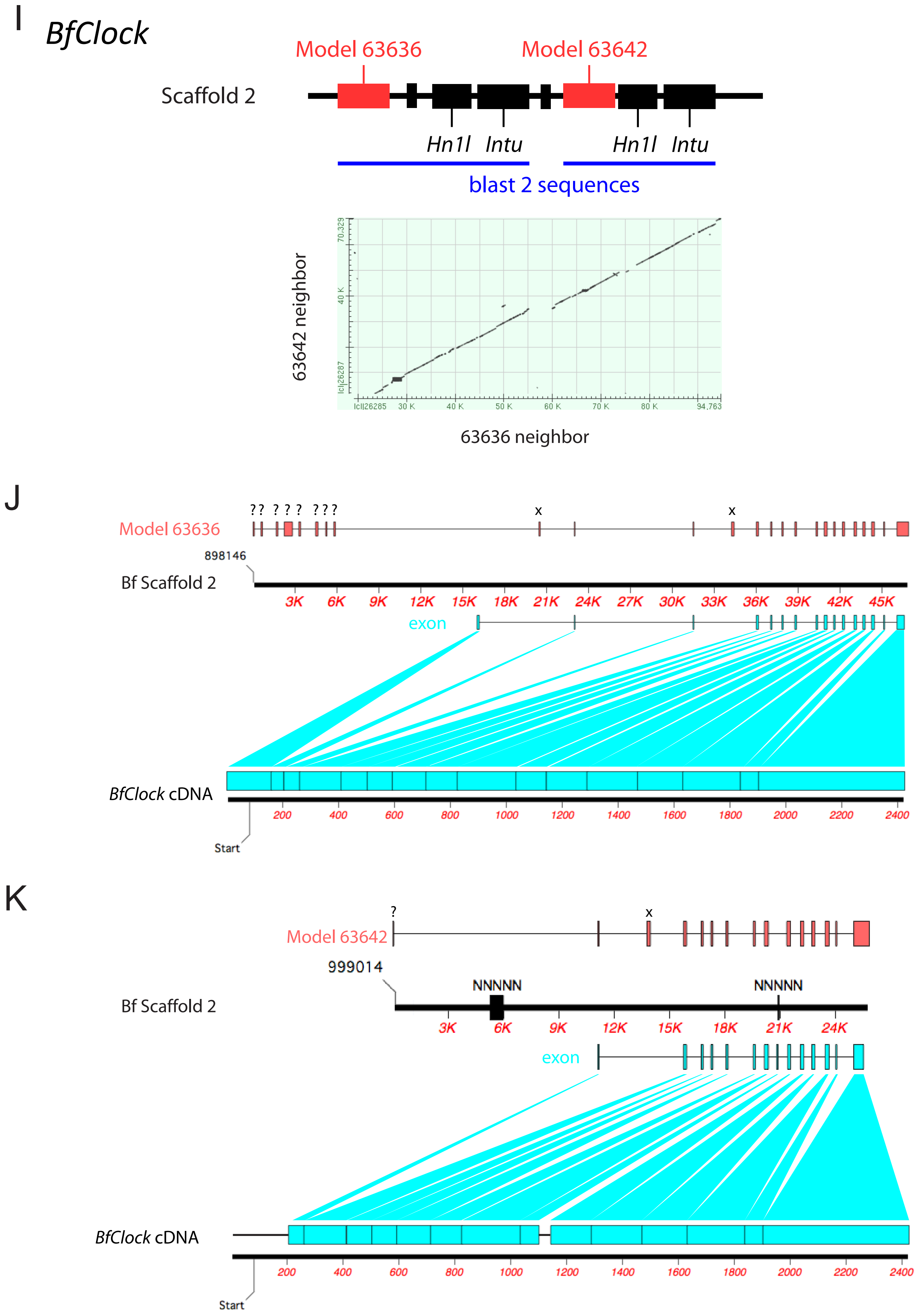
I
BfClock
Model 63636
Model 63642
Scaffold 2
Hn1l
Intu
Hn1l
Intu
blast 2 sequences
63642 neighbor
63636 neighbor
70,329
40 K
lcl|26287
lcl|26285
30 K
40 K
50 K
60 K
70 K
80 K
94,763
J
Model 63636
?? ??? ???
x
x
898146
Bf Scaffold 2
3K 6K 9K 12K 15K 18K 21K 24K 27K 30K 33K 36K 39K 42K 45K
exon
BfClock cDNA
200 400 600 800 1000 1200 1400 1600 1800 2000 2200 2400
Start
K
Model 63642
?
x
999014
Bf Scaffold 2
NNNNN
NNNNN
3K 6K 9K 12K 15K 18K 21K 24K
exon
BfClock cDNA
200 400 600 800 1000 1200 1400 1600 1800 2000 2200 2400
Start

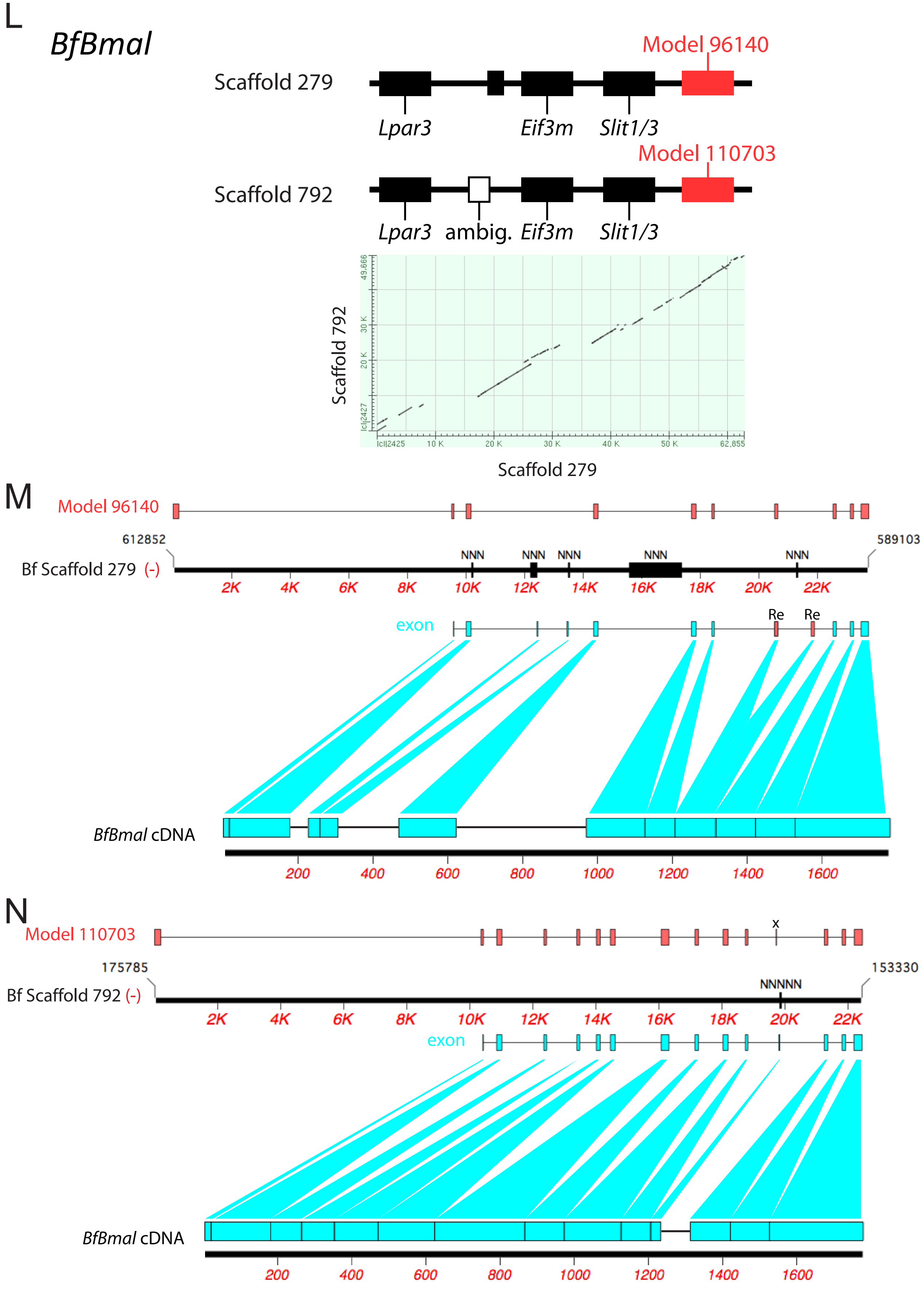
L
BfBmal
Scaffold 279
Model 96140
Lpar3
Eif3m
Slit1/3
Model 110703
Scaffold 792
Lpar3
ambig.
Eif3m
Slit1/3
Scaffold 792
49,666
30 K
20 K
lcl|2427
lcl|2425
10 K
20 K
30 K
40 K
50 K
62,855
Scaffold 279
M
Model 96140
612852
589103
Bf Scaffold 279 (-)
NNN NNN NNN NNN NNN
2K 4K 6K 8K 10K 12K 14K 16K 18K 20K 22K
exon
Re Re
BfBmal cDNA
200 400 600 800 1000 1200 1400 1600
N
Model 110703
x
175785
153330
Bf Scaffold 792 (-)
NNNNN
2K 4K 6K 8K 10K 12K 14K 16K 18K 20K 22K
exon
BfBmal cDNA
200 400 600 800 1000 1200 1400 1600

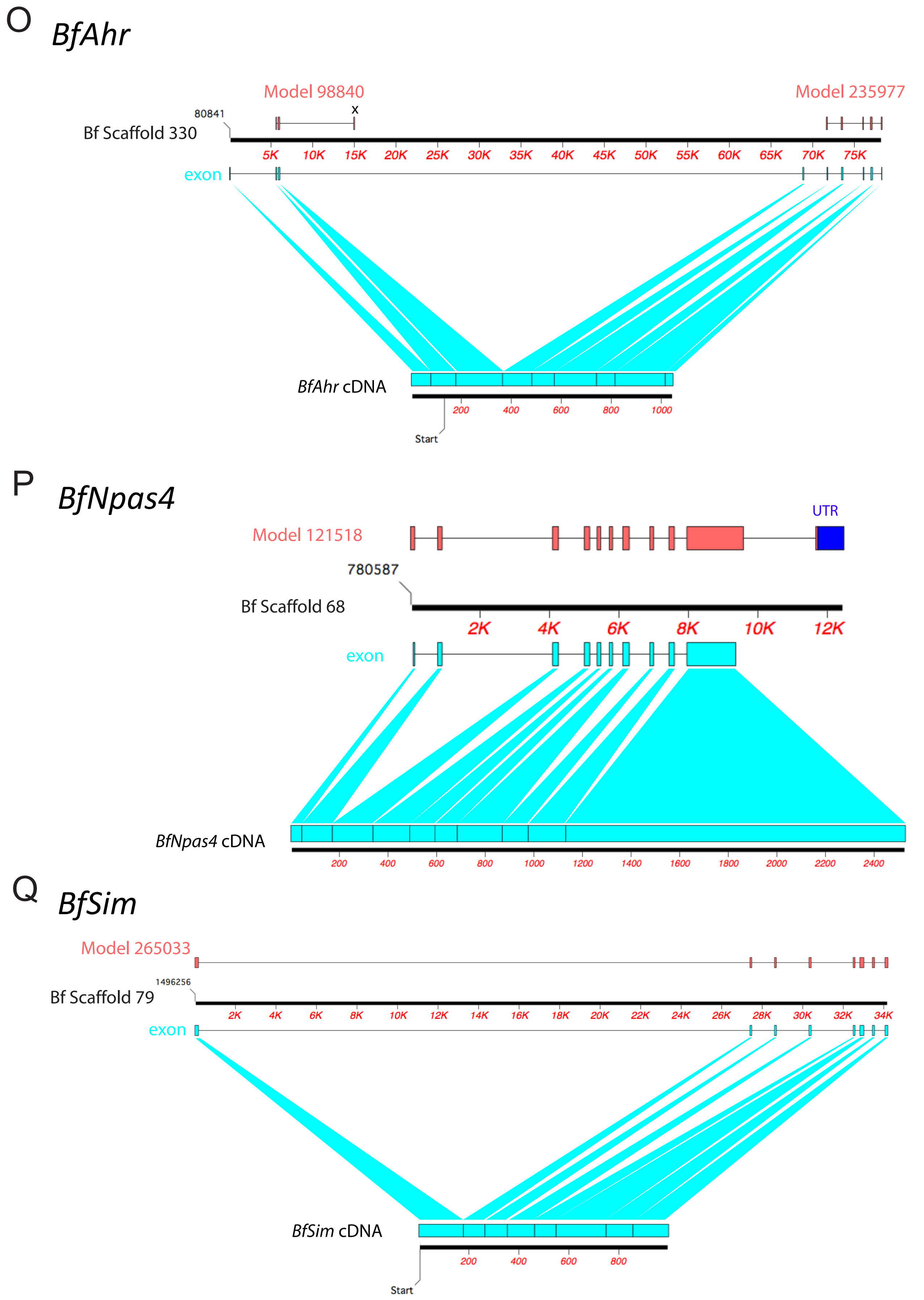

O
BfAhr
Model 98840
Model 235977
80841
x
Bf Scaffold 330
5K
10K
15K
20K
25K
30K
35K
40K
45K
50K
55K
60K
65K
70K
75K
exon
BfAhr cDNA
200
400
600
800
1000
Start
P
BfNpas4
UTR
Model 121518
780587
Bf Scaffold 68
2K
4K
6K
8K
10K
12K
exon
BfNpas4 cDNA
200
400
600
800
1000
1200
1400
1600
1800
2000
2200
2400
Q
BfSim
Model 265033
1496256
Bf Scaffold 79
2K
4K
6K
8K
10K
12K
14K
16K
18K
20K
22K
24K
26K
28K
30K
32K
34K
exon
BfSim cDNA
200
400
600
800
Start

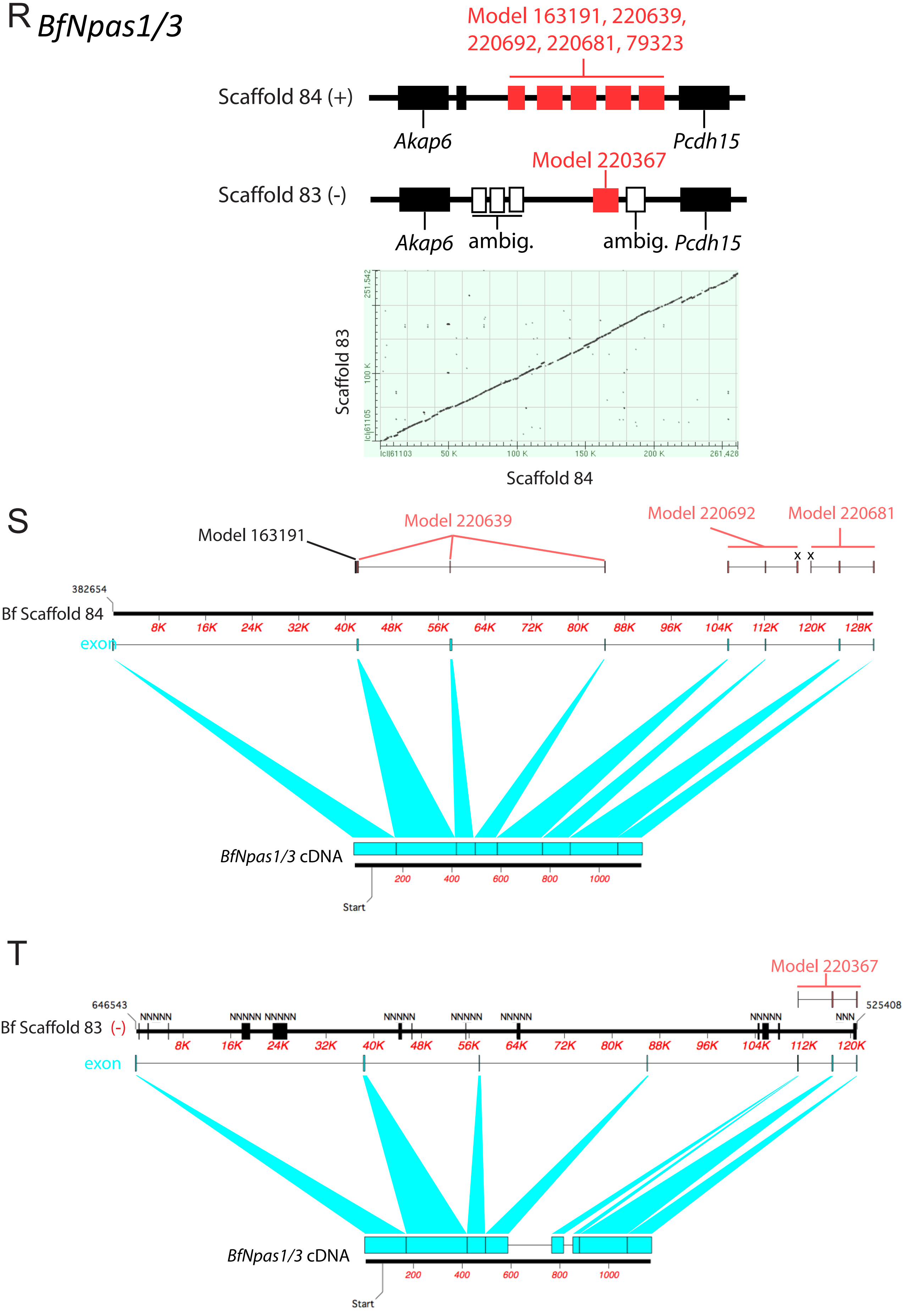

R
BfNpas1/3
Model 163191, 220639, 220692, 220681, 79323
Scaffold 84 (+)
Akap6
Pcdh15
Model 220367
Scaffold 83 (-)
Akap6
ambig.
ambig.
Pcdh15
Scaffold 83
Scaffold 84
S
Model 163191
Model 220639
Model 220692
Model 220681
382654
Bf Scaffold 84
8K 16K 24K 32K 40K 48K 56K 64K 72K 80K 88K 96K 104K 112K 120K 128K
exon
BfNpas1/3 cDNA
200 400 600 800 1000
Start
T
Model 220367
646543
525408
Bf Scaffold 83 (-)
NNNNN
NNN
8K 16K 24K 32K 40K 48K 56K 64K 72K 80K 88K 96K 104K 112K 120K
exon
BfNpas1/3 cDNA
200 400 600 800 1000
Start

Relationships of obtained cDNA, *B. floridae* genomic scaffolds, and gene models of *bHLH-PAS* genes.

In panels showing cDNA and genomic scaffolds (A, B, D, E, G, H, J, K, M-Q, S, and T), the upper black band with coordinates represents part of the genomic scaffold, with scaffold number labeled. Some scaffolds are shown in minus direction. Gene models are shown in red segmented boxes above the genomic scaffolds. The lower black band represents obtained cDNA of each *bHLH-PAS* genes. Putative start and stop codons are labeled. Presumed exons are shown as cyan segmented boxes below the genomic scaffolds. Ambiguous gap regions, which were not sequenced and denoted as strings of "Ns" in the genome browser, are shown as black boxes in E, K, M, N, and T. cDNA regions that cannot be aligned may be due to these regions. Exons predicted in gene models but not present in cDNA are labeled with "x". Predicted exons lacking evidence of its existence are labeled with "?". In the comparisons of redundant models (panels C, F, I, L, and R), the upper schematic figures (drawn not to scale) show positional relationships of *bHLH-PAS* gene models (red boxes) and their neighboring gene models (black boxes), and the lower X-Y plots show the comparisons of sequences. In the schematic figures, ambiguous gap regions are shown as open boxes (labeled with "ambig.") in scaffold 188, 792, and 83. The lower X-Y plots were made from comparing two scaffold regions showed above; compared regions are denoted by blue lines in C and I. The arrangement of gene models and the sequence similarity shown by comparison support that these two scaffolds or two scaffold regions are highly similar and may be one of the following: (1) two different haplotypes assembled separately, (2) mis-assembly of single region/allele, or (3) a product of local duplication occurred recently.

(A) The comparison of *BfNcoa* cDNA, the corresponding genomic scaffold, and gene model 212110. Five exons (2 upstream of and 3 downstream of the model) are not predicted in the model 212110, and the presumable start and stop codons of obtained cDNA are not predicted. (B) The comparison of *BfArnt* cDNA, the corresponding genomic scaffold, and gene model 124387. (C-E) The comparison of *BfHifα* cDNA and the two corresponding genomic regions. (C) The scaffold 35 contains two highly similar regions: both regions contain gene models of *BfHifα*, zinc finger protein (*Zfp808*, *Zfp112*, and *ZNF**, not sure of its assignment) and models of kelch-like proteins. The comparison of *BfHifα* cDNA, genomic scaffold regions, and gene model 208339 (D) or gene model 208408 (E) are shown. (F-H) The comparison of *BfbHLHPAS-orphan* cDNA and the two corresponding genomic regions. (F) Two regions neighboring the *BfbHLHPAS-orphan* gene models both contain gene models of *Ube3a* and *Trim23* genes. The comparison of *BfbHLHPAS-orphan* cDNA, genomic scaffolds, and gene model 117200 (G) or gene model 125569 (H) are shown. The blue box in H is predicted UTR, a presumed correct prediction. (I-K) The comparison of *BfClock* cDNA and the two corresponding genomic regions. (I) Two regions in the scaffold 2 show high similarity: the regions downstream of each *BfClock* model both have models representing *Hn1l* and *Intu* genes. The comparison of *BfClock* cDNA, genomic scaffolds, and gene model 63636 (J) or gene model 63642 (K) are shown. (L-N) The comparison of *BfBmal* cDNA and the two corresponding genomic regions. (L) Regions in two scaffolds show high similarity: both scaffold 279 and 792 have models representing *Lpar3*, *Eif3m* and *Slit1/3* genes just upstream of each *BfBmal* model. Some ambiguous regions in scaffolds 792 may contribute to the discrepancy between two scaffolds. The comparison of *BfBmal* cDNA, genomic scaffolds, and gene model 96140 (M) or gene model

110703 (N) are shown. In M, two redundant regions of scaffold 279 (labeled with “Re”) gave similar scores in comparison by BLAST (align two sequences), while the Spidey program suggests that the first region (coordinate: 592314-592206) be the exon. (O) The comparison of *BfAhr* cDNA, the corresponding genomic scaffold, and gene models 98840 and 235977. We suggest the two models, 98840 and 235977, should be joined together for a better model of *BfAhr* gene. (P) The comparison of *BfNpas4* and the corresponding genomic scaffold and gene model 121518. The blue box is the predicted UTR. (Q) The comparison of *BfSim* and the corresponding genomic scaffold and gene model 265033. (R-T) The comparison of *BfNpas1/3* cDNA and the two corresponding genomic regions. (R) Two scaffold regions in scaffold 84 and 83 containing *BfNpas1/3* model show high similarity: both regions have gene models representing *Akap6* and *Pcdh15* genes. Many ambiguous regions in scaffolds 83 may contribute to the discrepancy between two scaffolds. The comparison of *BfNpas1/3* cDNA, genomic scaffolds, and gene models on scaffold 84 (S) or gene model 220367 (T) are shown. In panel S, The model 163191 and the first exon of the model 220639 should be joined as a single exon, and four models showed should be joined to represent a single gene. The first exon including the presumable start codon of the *BfNpas1/3* cDNA is not predicted.
